# The concerted change in the distribution of cell cycle phases and zone composition in germinal centers is regulated by IL-21

**DOI:** 10.1038/s41467-021-27477-0

**Published:** 2021-12-09

**Authors:** Dimitra Zotos, Isaak Quast, Connie S. N. Li-Wai-Suen, Craig I. McKenzie, Marcus J. Robinson, Andrey Kan, Gordon K. Smyth, Philip D. Hodgkin, David M. Tarlinton

**Affiliations:** 1 Department of Immunology and Pathology, Monash University, 89 Commercial Road, Melbourne, VIC 3004 Australia; 2 The Walter and Eliza Hall Institute of Medical Research, 1G Royal Parade, Parkville, VIC 3052 Australia; 3 Department of Medical Biology, University of Melboure, Parkville, VIC 3010 Australia; 4 School of Mathematics and Statistics, University of Melboure, Parkville, VIC 3010 Australia; 5 Present Address: School of Computer Science, University of Adelaide, Frome Rd, Adelaide, SA 5005 Australia

**Keywords:** Cytokines, Germinal centres, B cells

## Abstract

Humoral immune responses require germinal centres (GC) for antibody affinity maturation. Within GC, B cell proliferation and mutation are segregated from affinity-based positive selection in the dark zone (DZ) and light zone (LZ) substructures, respectively. While IL-21 is known to be important in affinity maturation and GC maintenance, here we show it is required for both establishing normal zone representation and preventing the accumulation of cells in the G1 cell cycle stage in the GC LZ. Cell cycle progression of DZ B cells is unaffected by IL-21 availability, as is the zone phenotype of the most highly proliferative GC B cells. Collectively, this study characterises the development of GC zones as a function of time and B cell proliferation and identifies IL-21 as an important regulator of these processes. These data help explain the requirement for IL-21 in normal antibody affinity maturation.

## Abstract (web summary)

How IL-21 functions during development of high affinity antibody in germinal centres (GC) is not fully known. Here using a cell cycle reporter mouse, the authors show that IL-21 promotes cell cycle progression within the GC light zone and enables release from the G1 cell cycle stage.

## Introduction

Germinal centers (GC), formed in secondary lymphoid organs within days of infection or immunization, function to increase the affinity of responding B cells to the immunizing antigen^1–3^. This is achieved through iterative rounds of B cell proliferation, immunoglobulin (Ig) V-gene mutation, and the positive selection of clones with improved affinity for antigen. The differentiation of GC B cells throughout the reaction generates populations of high affinity, long-lived plasma cells (LLPC), and affinity-enriched memory B cells (MBCs), both hallmarks of T cell-dependent immune responses^1,3,4^. After a period of rapid clonal expansion, the number of GC B cells peaks and then declines to pre-immune response values over the ensuing weeks to months as cell loss through differentiation and death exceeds the generation of new cells^5–7^.

GC are established by rapid B cell proliferation, followed by the development of two distinct zones, which is essential for efficient affinity maturation and maintenance of the GC^1,2,6^. One of these zones, the light zone (LZ), is comprised of centrocytes (CC), centered on CXCL13-secreting follicular dendritic cells (FDC) and enriched also for the CD4 T follicular helper (Tfh) cells that are absolutely required for GC function. The adjacent dark zone (DZ), proximal to the T cell area, is comprised of centroblasts (CB) that are centered on CXCL12^+^ reticular stromal cells. B cell affinity maturation relies on processes that are partitioned in the two zones and requires cycling of cells between zones with concomitant oscillation of phenotypes^8^. CC in the LZ bind and acquire antigen from depots on FDC, interact with Tfh and initiate cell division or differentiation, while CB in the DZ undergo extended proliferation and AID-mediated IgV gene mutation^9,10^. It is proposed that success in accessing antigen on FDC increases the probability of productive interactions of CC with cognate Tfh, which largely determines the fate of the B cell^11,12^. CC may be triggered into division with the transient upregulation of Myc and activation of mTOR to promote cell growth and anabolic metabolism^13–15^, accompanied by phosphorylation of transcription factor Foxo1 to repress the LZ gene expression program and maintain that of the DZ^16,17^. As part of the transition to CB, CC upregulate CXCR4, mediating their migration to the DZ^18,19^. Continued proliferation of CB in the DZ requires continued expression of Myc targets including the transcription factor AP4 (refs. ^13,20^), while CB persistence in the DZ requires the continued presence of pFOXO1 and mTOR activity^15,21^. Coincident with cessation of CB proliferation, a CC phenotype is restored including reduced CXCR4 and FOXO1, dephosphorylation of FOXO1, increased surface expression of the BCR and MHCII, and return to the LZ to compete anew for access to antigen^22,23^. CB with BCR genes irreversibly damaged by SHM undergo apoptosis^23,24^. CC may pause for an unknown period, differentiate into plasma cells (PC) or MBC, or re-enter division, all depending on the stimulus they receive.

In the cyclic re-entry model^25^, GC persistence is mediated by cell division balancing cell loss. However, the identity and relative contributions of the different signals delivered to CC in determining their immediate fate remain largely undefined. Interleukin-21 (IL-21) secreted by Tfh is an important modulator of the B cell response to antigens, including proteins, parasites and viruses, both in mice and humans^26–29^. In several mouse studies, absence of IL-21 (*Il21*^−/−^) or its receptor (*Il21r*^−/−^) resulted in premature termination of the GC reaction with diminished proliferation, increased early MBC differentiation, reduced frequency of bone marrow plasma cells, and reduced affinity maturation^30–32^. Why *Il21r*^−/−^ GC B cells proliferate less than controls and how this relates to the immune abnormalities remains uncertain.

In this work, to clarify issues of GC B cell behavior and the contribution of IL-21, we undertook a detailed analysis of B cell proliferation during GC formation in response to protein immunization and assess the consequences of IL-21 deficiency. We quantify cell cycle phases in the GC and its zones using cell cycle indicator mice and thymidine analog incorporation, which shows unique and important functions for IL-21. We conclude that IL-21 promotes proliferation through entry into the S phase and thus functions to limit B cell time out of cell cycle. This action is required to establish a normal GC zone distribution and we propose this controls the duration of the reaction, the extent of affinity maturation, and the quality of GC output.

## Results

### IL-21 promotes entry into the cell cycle in GC B cells

**Fig. 1:**
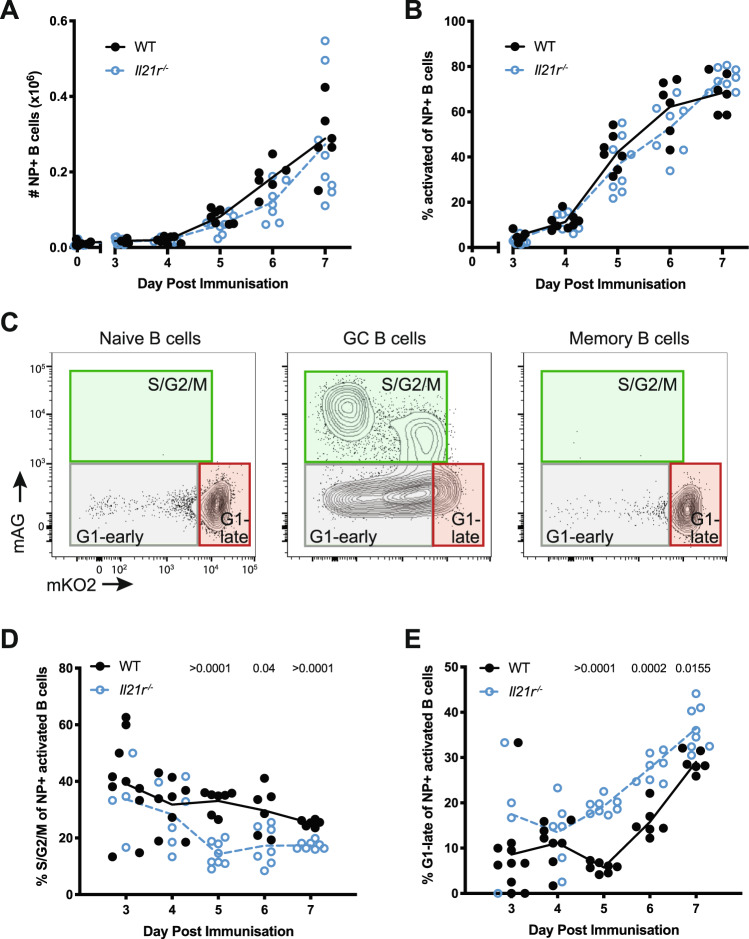
IL-21 promotes proliferation in GC B cells. **A** WT or *Il21r*^−/−^ mice were either unimmunized (day 0) or immunized i.p. with NP-KLH in alum and spleens analyzed 3, 4, 5, 6. and 7 days thereafter. The total number of NP-binding B cells (CD138^−^CD19^+^NP^+^) among total spleen lymphocytes is shown. **B** The frequency of early activated cells (Fas^+^CD38^−^) among NP-binding B cells shown in **A**. **C** Representative mKO2 and mAG expression profiles on splenocytes of a mouse 7 days post immunization with NP-KLH in alum, depicting naive (CD19^+^IgD^+^), GC (IgD^−^CD19^+^FAS^+^CD38^−^NP^+^), and memory (IgM^−^CD19^+^CD38^+^Fas^−^) B cells. Green (mAG^+^), gray (mAG− mKO2lo/−) and red (mKO2^hi^) boxes depict electronic gates used to identify cells in S/G2/M, G1-early, and G0 or G1-late, respectively. **D**, **E** Antigen-binding, activated B cells from NP-KLH in alum immunized WT and *Il21r*^−/−^ Fucci mice analyzed for proportion of cells in S/G2/M (**D**) and G1-late (**E**). Data in **A** and **B** represent three independent experiments pooled, with a total of 6–12 mice per time point. Data in **D** and **E** represent *n* = 10, 4 (WT, *Il21r*^−/−^; Day 3), *n* = 8, 6 (WT, *Il21r*^−/−^; Day 4), *n* = 7, 8 (WT, *Il21r*^−/−^; Day 5), *n* = 6, 7 (WT, *Il21r*^−/−^; Day 6), and *n* = 6, 8 (WT, *Il21r*^−/−^; Day 7) mice examined over three independent experiments. Only mice with ≥10 NP-binding GC B cells were considered for analysis. Each circle represents an individual mouse, lines connect means. Statistical significance determined by *t-*test and corrected for multiple comparison using the Holm–Sidak method. Exact *p* values are shown with those ≤0.05 considered significant. Source data are provided as a Source Data file.

Control (WT) and *Il21r*^−/−^ mice were immunized by intraperitoneal (i.p.) injection with the T cell-dependent antigen (4-hydroxy-3-nitrophenyl)acetyl coupled to keyhole limpet hemocyanin (NP-KLH) in alum and the number of NP-binding B cells in the spleen measured as a function of time (Fig. 1A and Supplementary Fig. 1A, B). The number of NP^+^ B cells was significantly above pre-immunization values by day 3 in both strains and increased further over the next 4 days of the analysis (Fig. 1A). The proportion of NP-specific B cells expressing prototypical GC surface markers increased in parallel in both strains from day 3, reaching approximately 70% by day 7 (Fig. 1B). Previous studies by us and others identified potential differences in B cell proliferation of *Il21*^−/−^ and *Il21r*^−/−^ mice after immunization^31,32^. To investigate further this possibility, we used the fluorescence ubiquitination cell cycle indicator (FUCCI) system^33^ in which the phase-specific degradation of two FUCCI reporter proteins—monomeric Kusabira orange (mKO2) and monomeric Azami green (mAG)—allowed identification and enumeration of cell cycle distribution by flow cytometry (Supplementary Fig. 1C). Analyzing follicular B cells, GC B cells, and MBC of WT FUCCI mice day 7 after NP-KLH immunization attested to the applicability of the dual FUCCI reporter system to B cells and GC (Fig. 1C and Supplementary Fig. 1D), confirming other studies^23,34^. Spleen GC B cells (CD19^+^IgD^−^ FAS^+^ NP^+^ CD38^−^) from day 7 immunized WT mice displayed all cell cycle phases while MBC, identified as CD19^+^IgM^−^Fas^−^CD38^+^, contained high mKO2 (Cdt1 fused; G0/G1) and little if any mAG (Geminin fused; S/G2/M), similar to naive B cells and consistent with cessation of proliferation. For GC B cells, all of which were Ki67^+^ and therefore not in G0 (Supplementary Fig. 2), we defined three populations, depicted in Fig. 1C, consistent with other pubications^23^: (1) cells expressing any amount of mAG, indicating S/G2/M; (2) cells expressing low to intermediate amounts of mKO2, indicating early G1; and (3) cells expressing high amounts of mKO2, indicating late G1. Resolving early and late G1 by amounts of mKO2 reflects its accumulation in cells with time out of S/G2/M^35^ (Supplementary Fig. 1).

To investigate the impact of IL-21 signaling deficiency on the cell cycle, we generated *Il21r*^−/−^ FUCCI mice, immunized them with NP-KLH in alum and analyzed the B cell response over time, in comparison to the matched WT mice. The frequency of antigen specific, GC B cells (NP^+^CD19^+^CD38^−^FAS^+^) in S/G2/M in WT mice at day 3 after immunization was variable but averaged around 40%, but declined over the next 4 days to be 25% of NP^+^ GC B cells by day 7 (Fig. 1D). The proportion of NP^+^ GC B cells in S/G2/M in *Il21r*^−/−^ mice was consistently lower than WT, dropping to and then remaining at <20% from day 5 to 7 (Fig. 1D). In contrast, the proportion of NP^+^ GC B cells in late G1 was higher in *Il21r*^−/−^ than in WT immunized mice on all days, reaching statistical significance on days 5, 6, and 7 (Fig. 1E) when 20, 30, and 35% of NP^+^ GC B cells had become mKO2 high in *Il21r*^−/−^ mice, compared to 5, 15, and 30% in WT. Tfh cell number during this period was not significantly different between strains, making this an unlikely explanation (Supplementary Fig. 1). From these data we concluded that IL-21 inhibited the accumulation of GC B cells in the late G1 phase of the cell cycle over the first week of a response.

### IL-21 mediates the kinetics of GC zone formation

**Fig. 2:**
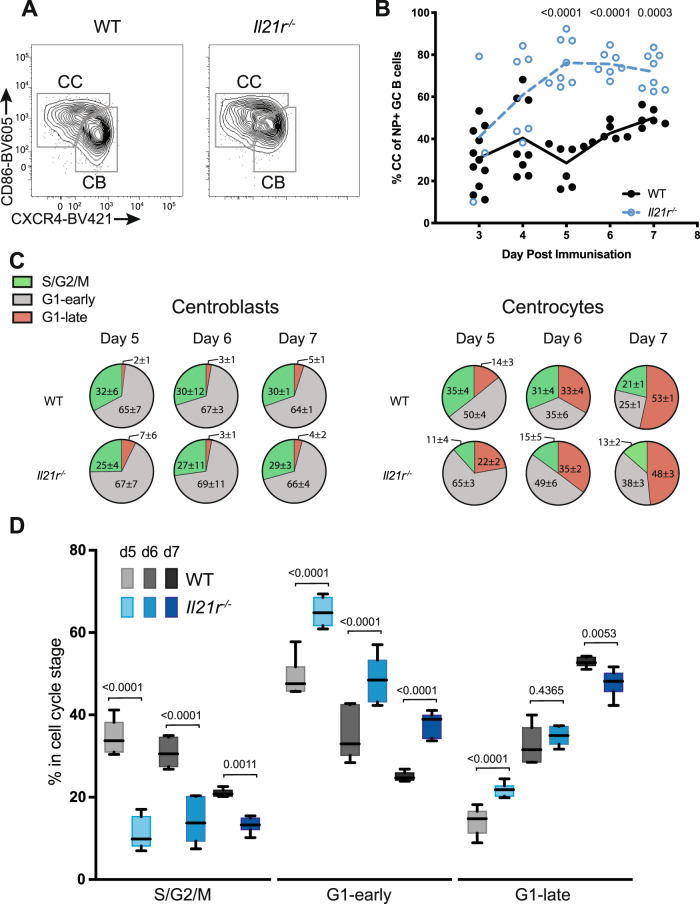
GC zone development and composition is affected by IL-21. **A** WT and *Il21r*^−/−^ mice were immunized i.p. with NP-KLH in alum and spleen NP-binding GC B cells (CD138^−^CD19^+^NP^+^CD38^−^Fas^+^) analyzed for CD86 and CXCR4 expression to identify light zone centrocytes (CC; CD86^hi^CXCR4^lo^) and dark zone centroblasts (CB; CD86^lo^CXCR4^hi^). Representative day 7 flow cytometric gating of GC zone division is shown for WT (left) and *Il21r*^−/−^ (right) mice. **B** The frequency of antigen-specific GC B cells with light zone (CD86^hi^CXCR4^lo^) phenotype as a function of time after immunization. Pooled data represent *n* = 11, 3 (WT, *Il21r*^−/−^; Day 3), *n* = 8, 6 (WT, *Il21r*^−/−^; Day 4), *n* = 7, 8 (WT, *Il21r*^−/−^; Day 5), *n* = 6, 7 (WT, *Il21r*^−/−^; Day 6), and *n* = 6, 8 (WT, *Il21r*^−/−^; Day 7) mice examined over three independent experiments. Each circle represents an individual mouse, with solid for WT and open for *Il21r*^−/−^. Statistical significance determined by *t-*test and corrected for multiple comparison using the Holm–Sidak method. Exact *p* values are shown with those ≤0.05 considered significant. **C** Cell cycle phase distribution determined by FUCCI profiles as described in Fig. 1 and shown as the percentages of the total dark zone (CB; left) or light zone (CC; right) NP-specific GC B cells from WT and *Il21r*^−/−^ splenocytes at time points post immunization indicated. Pie charts show mean ± SD at indicated days after immunization with green, gray and red depicting proportions of cells in S/G2/M, G1-early, and G1-late, respectively. **D** Statistical analysis light zone (CC) cell cycle phase distribution. **C**, **D** Data represent *n* = 7, 8 (WT, *Il21r*^−/−^; Day 5), *n* = 6, 7 (WT, *Il21r*^−/−^; Day 6), and *n* = 6, 8 (WT, *Il21r*^−/−^; Day 7) mice examined over three independent experiments. Only mice with ≥10 NP-binding light zone or dark zone GC B cells were considered for analysis and are not counted in *n* number stated here. Boxes depict 25th to 75th percentiles with the line showing the median. Whiskers show minimal and maximal values with gray boxes depicting data from WT and blue *Il21r*^−/−^ mice, respectively. Statistical significance in **D** was determined by two-way ANOVA and Sidak’s multiple comparisons test. Exact *p* values are shown with those ≤0.05 considered significant. Source data are provided as a Source Data file.

We reasoned that a deficit in B cell proliferation in *Il21r*^−/−^ mice early after immunization might affect GC zone formation, especially as differences have been noted in late stages of the immune response of *Il21r*^−/−^ mice^26,36^. We examined GC B cell zone distribution in WT and *Il21r*^−/−^ mice following immunization, using amounts of CD86 and CXCR4 to identify CC (CD86^hi^, CXCR4^lo^) and CB (CD86^lo^, CXCR4^hi^) phenotype cells^37^ (Fig. 2A). At day 3, approximately 30% of WT NP^+^ GC B cells had a CC phenotype (Fig. 2B). Despite the number of NP^+^ GC B cells increasing (Fig. 1B) in WT mice, the CC proportion remained constant through to day 6 at 40% and then increased to 50% on day 7. In comparison, the proportion of *Il21r*^−/−^ GC B cells with CC phenotype was variable on days 3–4, then consolidated at, and remained greater than, 70% from day 5 to 7, significantly increased over WT (Fig. 2B). Immunization of mixed chimeras, created by reconstitution of irradiated recipients with a 50:50 mixture of WT and *Il21r*^−/−^ bone marrow, revealed the CC bias to be intrinsic to B cell loss of the receptor (Supplementary Fig. 3), reinforcing the independence of this phenotype from Tfh cells or Tfr cells. Collectively these data showed LZ to develop over a limited time in WT GC in a process that was significantly impeded by IL-21 signaling, while reciprocally, the expansion of DZ phenotype B cells was significantly accelerated by IL-21 signaling.

We next examined GC B cells in the different zones for cell cycle phase distribution as revealed by FUCCI, using gates depicted in Figs. 1C and 2A. Perhaps surprisingly, given the reduced frequency of CB in *Il21r*^−/−^ mice, the distribution of cell cycle phases among NP-specific CB was constant over time and indistinguishable from WT (Fig. 2C, left). From day 5 to 7, approximately 30% of both WT and *Il21r*^−/−^ CB were in S/G2/M and 2–7% in late G1 (Fig. 2C). The cell cycle phase distribution in CC, however, varied between strains and time points (Fig. 2C, D). The proportion of CC in the S/G2/M phase of the cell cycle in *Il21r*^−/−^ mice averaged 11–15% from day 5 to 7, significantly less than in their WT counterparts that declined—from 35 to 21%—over the same interval (Fig. 2C, right and D). While the proportion of CC in early G1 declined in both strains from day 5 to 7, it was significantly higher in *Il21r*^−/−^ than WT at each time: 65–38% in *Il21r*^−/−^ compared with 50–25% in WT. The LZ of both WT and *Il21r*^−/−^ mice contained a larger fraction of cells in late G1 than did their respective DZ, and this increased in both strains from day 5 values of 14% (WT) and 22% (*Il21r*^−/−^) to be approximately 50% in each at day 7 (Fig. 2C, D). These data revealed roles for IL-21 in establishing the representation of the DZ and LZ and the proliferation profile of CC, particularly in the distribution of cells synthesizing DNA or having just completed the cell cycle.

We asked whether the abnormality in zone distribution seen with IL-21 deficiency occurred also in the absence of IL-4 signaling in GC B cells. We immunized mice previously irradiated and reconstituted with a 50:50 mixture of bone marrow WT and *Stat6*^−/−^ donors, marked respectively by expression of CD45.1 and CD45.2, and analyzed NP-specific GC B cells 7 days later. This revealed a significant reduction in representation of STAT6-deficient B cells in GC, but a normal LZ:DZ ratio among those that were present (Supplementary Fig. 3E). That is, establishing a normal ratio of GC zones depended on signals provided by IL-21 was not only a consequence of abnormal GC B cell expansion.

### Cell cycle phases are distributed across GC zone phenotypes

**Fig. 3:**
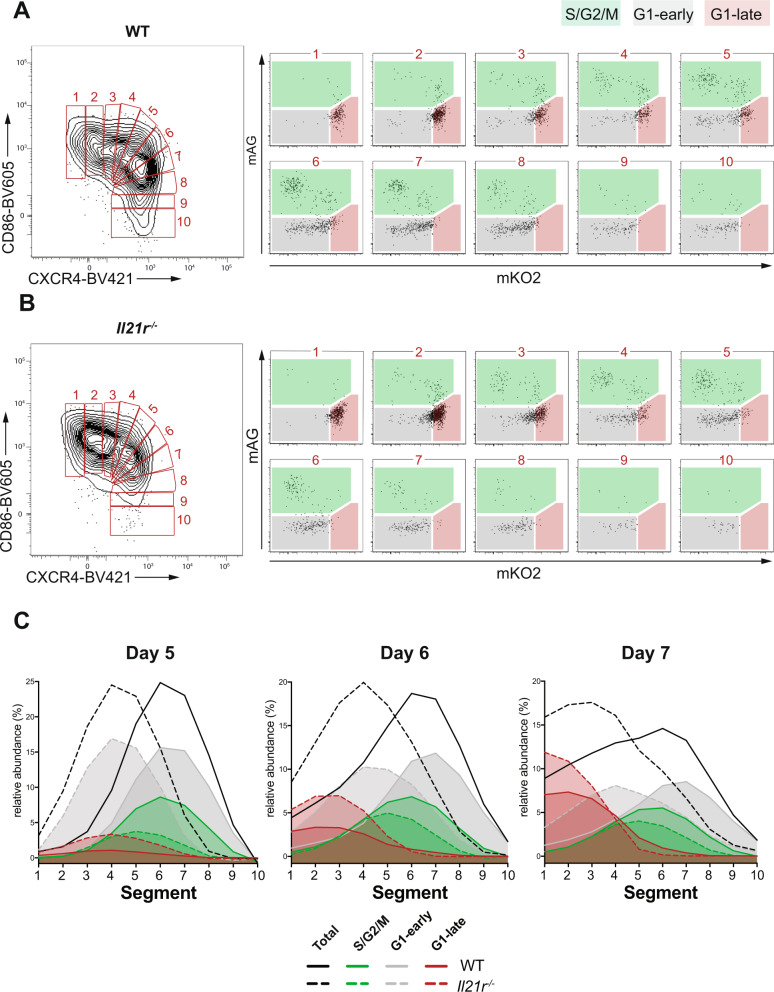
IL-21 deficiency alters cell cycle phase distribution across GC zones. Representative flow cytometry profile of day 7 splenic WT (**A**) and *Il21r*^−/−^ (**B**) NP-binding GC B cells (CD138^−^CD19^+^NP^+^CD38^−^Fas^+^) showing the 10 electronic gates (segments) used to analyze Fucci reporter profile in relation to CXCR4 and CD86 expression (left, large graphs) and the corresponding mKO2 vs mAG profiles (right, small graphs) with gates to identify S/G2/M (green shaded), G1-early (gray shaded), and G1-late (red shaded). **C** Relative abundance of total cells (black lines) and cells in individual cell cycle phases (shaded histograms) among the segments indicated in **A** and **B** at days 5, 6, and 7 post immunization. Solid lines indicate WT and dashed lines *Il21r*^−/−^. Curves were interpolated using GraphPad Prism 7 and 8. Data represent three independent experiments with 6, 7, or 8 mice per time point per genotype; final gates with ≤10 events were excluded. Source data are provided as a Source Data file.

As CXCR4 is crucial for dark zone location and CD86 is involved in T:B cell interaction, the relative expression of these two molecules may correlate with cells at different cell cycle phases and those in transit between LZ and DZ. To examine the relationship between cell cycle distribution and the gradation of GC zone phenotypes, we partitioned WT and *Il21r*^−/−^ NP^+^ GC B cells on days 5, 6 and 7 post immunization into 10 arbitrary segments across the spectrum of CD86 and CXCR4 co-expression (Fig. 3A, B). Segments (S)1–4 corresponded to CD86^hi^ LZ cells with increasing CXCR4; S6–10 to CXCR4^hi^ DZ cells with decreasing CD86 and S5 to cells with CD86^hi^CXCR4^hi^, equivalent to the recently described gray zone (GZ)^38^. On each day we calculated the relative abundance of B cells in each segment and the distribution of their cell cycle phase using FUCCI reporter expression (Fig. 3A, B). As expected from the cell counts (Figs. 1 and 2), the peak of WT GC B cell distribution on each day was among DZ cells, but here revealed as those expressing the most CD86. B cells in *Il21r*^−/−^ GC were most frequent in the LZ, centered on intermediate expression of CXCR4 (Fig. 3C, quantification in Supplementary Fig. 4). Analysis of the late G1 cell cycle stage (mKO2^hi^) of NP^+^ GC B cells showed a gradual accumulation over time, which was accompanied by a phenotypic shift towards expressing the least CXCR4 (S1–2; Fig. 3); both parameters were markedly increased in the absence of IL-21 signaling. Cells in S/G2/M were most frequent in both strains at the transition between LZ and DZ phenotypes (S4–7) but sparse among remaining LZ cells and, perhaps surprisingly, DZ cells (S1–2 and S9–10, respectively; Fig. 3). Cells in the synthesis (S/G2/M) phase of the cell cycle were centered on S5–6 in both WT and *Il21r*^−/−^ on all days, revealing a concordance of zone phenotype and this phase of cell cycle that was independent of IL-21. In WT GC, the distribution of early G1 cells was similar to that of S/G2/M cells, albeit expressing slightly lower CD86 (Fig. 3C). In contrast, and in the biggest difference between strains, early G1 cells in *Il21r*^−/−^ GC were predominantly and consistently of a LZ phenotype, expressing less CXCR4 than their WT counterparts or their S/G2/M precursors (Fig. 3C). To facilitate integration of these data with published results^38^, we re-analyzed using LZ, GZ, and DZ gates (Supplementary Fig. 5A). As shown (Supplementary Fig. 5B), the LZ bias in *Il21r*^−/−^ cells was still accompanied by a loss of DZ cells, but the GZ proportion remained essentially unaffected. When overlaying FUCCI reporter expression, an overall reduction of cells in S/G2/M and a bias of early and late G1 cells towards the LZ was again apparent (Supplementary Fig. 5C). Overall, these results indicated that the distribution of cell cycle phases was altered in the absence of IL-21 and most notably in the LZ, notwithstanding the unchanged phenotype of the majority of proliferating cells.

We examined other parameters in relation to GC zone and cell cycle phases. Average cell size was distributed in parallel with S/G2/M (mAG^+^) cells, with the largest cells being in S5–7 for both strains while remaining LZ and DZ cells were equally smaller (S1–3 and S8–10; Supplementary Fig. 4). This was despite LZ cells with lowest CXCR4 being enriched for late G1 and the DZ cells with lowest CD86 comprised mostly of early G1 and S/G2/M cells. Increased CD38 expression, which has been used as a marker for memory precursor cells in GC^34^, was highest in LZ with lowest CXCR4 (S1–2), declining incrementally in both strains with increasing CXCR4 expression to reach about 50% maximum in cells transitioning between zones. Indeed, expression of CD38 more closely followed the distribution of mKO2 expressing cells—time in G1—than cell size alone (Supplementary Fig. 4).

### LZ B cells are less likely to enter division over time in the absence of IL-21 signaling

**Fig. 4:**
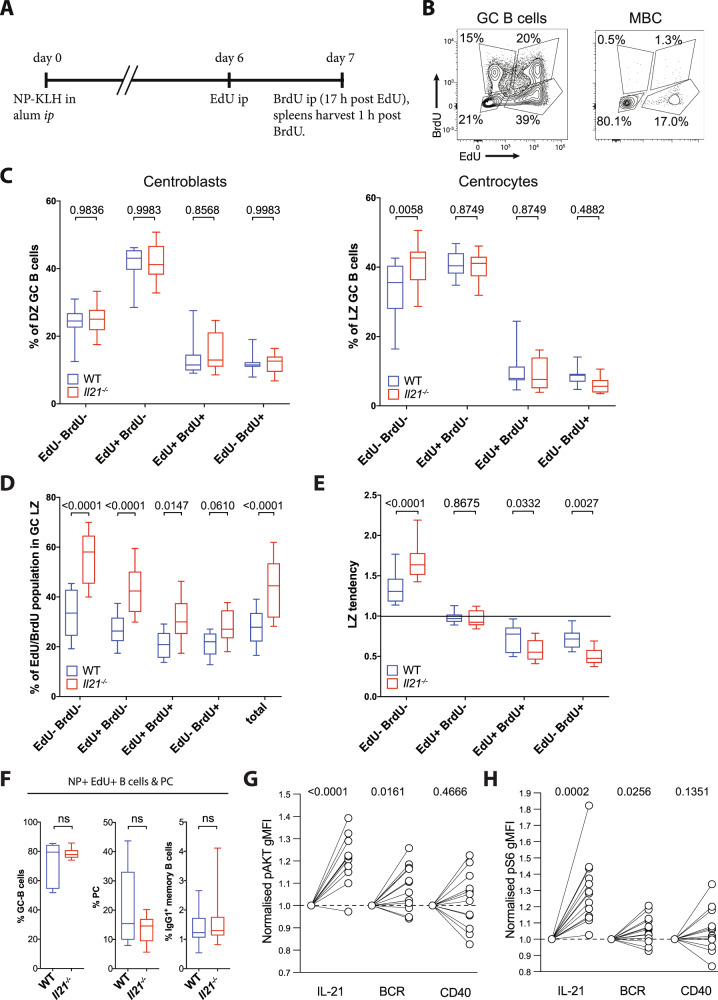
IL-21 deficiency affects GC LZ B cell proliferation over an extended period. **A** Experimental setup of NP-KLH immunization and thymidine analog (EdU and BrdU) injection of WT and *Il21*^−/−^ mice to analyze cell cycle phase progression over time. **B** Representative EdU vs BrdU profile of GC B cells and memory B cells (MBC) with gates to identify the four resultant populations indicated. See text for further details about the interpretation of EdU and BrdU populations. **C** NP-binding GC centroblasts (left) and centrocytes (right) analyzed for their composition of cells belonging to the four EdU/BrdU populations. **D** Proportion of LZ phenotype within each of the EdU/BrdU populations in WT and *Il21*^−/−^ GC B cells. **E** LZ tendency of EdU/BrdU populations after normalization for total LZ bias of total WT and *Il21*^−/−^ GC, respectively. **F** Occurrence of GC B cells, PC, and IgG1 memory B cells among total EdU+ NP-binding B cells and PC. **G** AKT and **H** S6 phosphorylation following ex vivo stimulation of GC B cells with IL-21, anti-Igκ/λ (BCR), or agonistic anti-CD40. Data represent *n* = 10 (WT) and *n* = 9 (*Il21r*^−/−^) biologically independent mice. Boxes depict 25th to 75th percentiles with the line showing the median. Whiskers show minimum and maximum values with WT in blue and *Il21r*^−/−^ in red. Statistical significance determined by *t-*test and corrected for multiple comparison using the Holm–Sidak method (**C**–**F**) or one-sample *T*-test (**G**, **H**). Exact *p* values are shown with those ≤0.05 considered significant. Source data are provided as a Source Data file.

We considered that measuring the proliferation history of GC B cells could provide further insights into the role of IL-21 signaling. To this end, we immunized WT and *Il21*^−/−^ mice with NP-KLH in alum and 6 days later injected them with the thymidine analog EdU to label cells actively synthesizing DNA. Seventeen hours later, sufficient time for any cell labeled during the EdU pulse to have completed that cycle and become available for re-entry, the mice were injected with BrdU and analyzed after 1 h (Fig. 4A). This protocol generated four populations of NP^+^ B cells: EdU^−^BrdU^−^ cells that were not in the S phase at the start of the experiment (between 18 and 15 h, EdU labeling window) or 1 h prior to analysis; EdU^+^BrdU^−^ cells that were in the S phase at the start of the experiment but not 1 h prior to analysis; EdU^+^BrdU^+^ cells that were in the S phase both at the start and again within 1 h of analysis; and EdU^−^BrdU^+^ cells that were in the S phase within 1 h but not at the start of analysis. These populations were exemplified using WT NP^+^FAS^+^CD38^−^ GC and MBCs (Fig. 4B and Supplementary Fig. 6). GC B cells from WT and *Il21*^−/−^ mice treated under this protocol were divided into CB or CC compartments using CXCR4 and CD86 expression and the representation of the four EdU/BrdU populations assessed (Fig. 4C and Supplementary Fig. 6). The distribution of DZ B cells among the four proliferation compartments was the same in both strains with the most populous being EdU^+^BrdU^−^, followed by EdU^−^BrdU^−^ and then the two BrdU^+^ fractions, which comprised cells recently in the S phase. The frequency of DZ cells in the S phase was about 15% in both strains, similar to the FUCCI results (Fig. 2C). The distribution of LZ B cells among the four fractions differed from that of DZ B cells in that EdU^−^BrdU^−^ and EdU^+^BrdU^−^ cells were equally represented by 35–45% of all LZ cells, while cells synthesizing DNA immediately prior to the assay (BrdU^+^) were approximately 15% in *Il21*^−/−^ compared to 25% in WT, values again consistent with FUCCI measurements on the same populations (Fig. 2C). This experiment revealed a significant excess of *Il21*^−/−^ LZ cells that had not synthesized DNA during either pulse compared to WT (Fig. 4C). We addressed whether recent proliferation was related to the zone distribution of GC B cells and influenced IL-21. The frequency of LZ cells within each of the four proliferation compartments was measured (Fig. 4D). *Il21*^−/−^ GC showed an over-representation of LZ cells in all compartments, some by almost twofold (Fig. 4D). Even accounting for the 1.5-fold expansion of the LZ in *Il21*^−/−^ and *Il21r*^−/−^ mice, EdU^−^BrdU^−^ cells remained significantly over-represented in *Il21*^−/−^ LZ compared to WT, while both EdU^+^BrdU^+^ and EdU^−^BrdU^+^ cells were significantly under-represented in *Il21*^−/−^ LZ (Fig. 4E). These results highlighted the role of IL-21 in initiating DNA synthesis in LZ B cells, as distinct to maintaining proliferation of cells that had already divided.

We tested a role for IL-21 in promoting cell division using an in vitro culture system to simulate GC^39^. After 48 h of stimulation with CD40L plus BAFF, B cell cultures were either pulsed or not with IL-21 for 2 h (Supplementary Fig. 7). One day later, the change in extent of B cell division with and without transient IL-21 exposure was measured by CTV dilution. B cells not exposed to IL-21 increased their mean division number to 1.9, while those pulsed with IL-21 progressed significantly further, and now averaged 2.3 divisions. Overall, IL-21 did not increase the maximum division number of the population but rather increased the proportion of cells progressing from one division to the next, increasing the fraction of cells in division 3 from 20 to 30% while diminishing that in division 1 from 23 to 13% (Supplementary Fig. 7D). Thus, in an in vitro system mimicking the GC^39^, transient exposure to IL-21 enhanced the proportion of B cells entering cell division, consistent with our in vivo observations.

An excess of MBC in the absence of IL-21 signaling has led to suggestions IL-21 may regulate their differentiation^31,32^. To assess the impact of IL-21 on GC B cell fate allocation, we assessed the phenotype of all EdU labeled NP^+^ B cells in WT and *Il21*^−/−^ mice, 18 h post pulse (Fig. 4F). We observed no significant differences between WT and *Il21*^−/−^ mice, with the majority of the EdU^+^ cells in both strains remaining as GC (approximately 80%), a significant minority being PC (approximately 15%) and a small fraction differentiating into IgG1 MBC (1%). The 18 h time frame was sufficient for MBC differentiation and acquisition of CD38 expression, as a sizeable fraction of the total MBC pool (identified as NP binding, IgG1^+^CD38^+^FAS^−^ B cells) incorporated EdU (Fig. 4B). Thus, the low MBC differentiation rate observed was not due to delayed upregulation of CD38 and an inability to identify MBC but rather reflected low MBC output at this stage of the response. While some EdU^+^ PC could have arisen from PB proliferating at the time of the pulse, the frequency of EdU^+^ MBC, which cease proliferation on or before formation, reflected only those GC B cells that were replicating DNA during the EdU pulse. That the fraction of labeled B cells that had become MBC in both strains was equal indicated that the frequency of this process was not significantly affected by the absence of IL-21.

To address how IL-21 influences GC B cells, we considered the possibility that it promotes pathways downstream of BCR or CD40 that result in cell proliferation. AKT phosphorylation, a key component of PI(3)K signaling, is attenuated in GC downstream of the BCR^40^ and has been reported to be induced by IL-21 (ref. ^41^), making it an attractive candidate by which IL-21 could influence population dynamics within GC. AKT acts to mediate cell cycle progression by phosphorylating S6 in an mTOR-dependent manner. To investigate this pathway, we immunized mice i.p. with NP-KLH and at day 7, harvested spleens and enriched GC B cells by magnetic sorting (Supplementary Fig. 8A). GC B cells were then incubated for 2 h with or without 20 ng IL-21 or agonistic anti-CD40 or BCR stimulation (anti-kappa and lambda light chain cross-linking antibodies). Phosphoflow analysis showed that IL-21 exposure increased phosphorylation of AKT (at S473) in GC B cells (Fig. 4G and Supplementary Fig. 8B). In line with previous results^40^, BCR signaling also resulted in some AKT phosphorylation whereas anti-CD40 did not alter pAKT amounts under the conditions tested (Fig. 4G and Supplementary Fig. 8B). Consistent with AKT’s role in promoting S6 phosphorylation, IL-21 and BCR stimulation but not anti-CD40 also resulted in increased pS6 (Fig. 4H and Supplementary Fig. 8B). Collectively, these results indicated that while IL-21 minimally influenced the phenotypic characteristics of GC B cells, it promoted proliferation of cells that would otherwise remain quiescent by, at least in part, increasing AKT and S6 phosphorylation.

### GC B cell transcriptomics reflects IL-21 impact on zones and proliferation

**Fig. 5:**
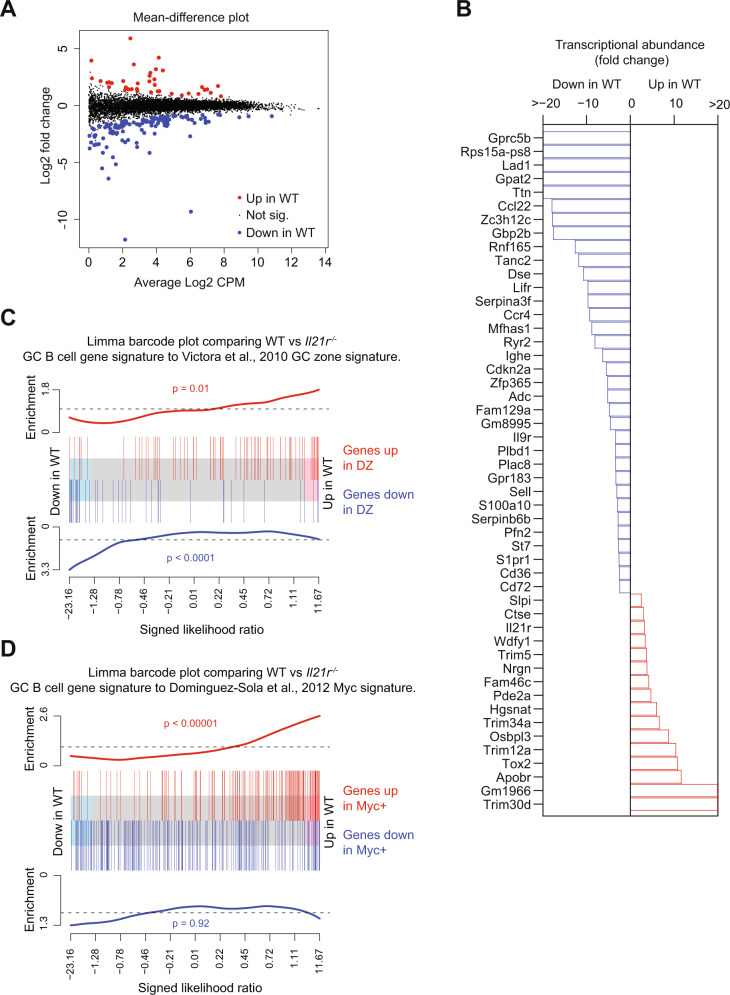
LZ bias in the absence of IL-21 signaling is confirmed by gene expression analysis. **A**–**C** WT and *Il21r*^−/−^ mice were immunized i.p. with NP-KLH in alum and on day 7, spleen NP-binding GC B cells (CD138^−^IgM^−^IgD^−^Gr1^−^B220^+^NP^+^IgG1^+^CD38^−^) were sorted and RNA-seq performed. **A** Mean-difference plot of WT vs *Il21r*^−/−^ GC B cells depicts genes that were significantly upregulated in WT compared to *Il21r*^−/−^ GC B cells shown in red and significantly downregulated ones in blue (false discovery rate <0.05). **B** Gene expression fold changes in WT compared to *Il21r*^−/−^ GC B cells. Significantly DE genes were ranked by FDR and the 50 lowest then ordered by FC, colored red if upregulated in WT compared to *Il21r*^−/−^, blue if downregulated. **C**, **D** limma barcode plots testing for GC zone signature or cMyc signature genes among DE genes from WT vs *Il21r*^−/−^ GC B cells as in **A**. Genes were ranked left to right as most up in *Il21r*^−/−^ to most up in WT GC. For zone signature (**C**), vertical red bars mark DZ signature genes while vertical blue bars mark LZ signature genes. Worms show relative enrichment of DZ and LZ signature genes and camera *p* values assess significance. The plot shows DZ genes enriched among those up in WT while LZ genes were enriched among those up in *Il21r*^−/−^ GC. For the Myc signature (**D**), vertical red bars mark upregulated genes in Myc^+^ cells while vertical blue bars mark genes upregulated in Myc^−^ cells. Worms show relative enrichment of Myc signature genes and camera *p* values assess significance. The plot shows Myc^+-^associated genes were significantly up in WT GC B cells.

We examined GC B cells for evidence of transcriptional changes caused by the absence of IL-21 signaling. NP-binding GC B cells (CD138^−^IgM^−^IgD^−^Gr1^−^B220^+^NP^+^IgG1^+^CD38^−^) were sorted from the spleens of WT and *Il21r*^−/−^ mice day 7 after immunization i.p. with NP-KLH in alum (Supplementary Fig. 8C). Analysis of RNA-seq data revealed 164 genes to be significantly differentially expressed (DE) in WT compared to *Il21r*^−/−^ GC B cells, with 126 up- and 38 down-regulated (Fig. 5A), the top 30 of each in both directions were plotted (Fig. 5B). None of the DE genes were previously identified as regulators of GC B cell behavior, such as *cMyc* or *Foxo1*, emphasizing the normality of *Il21r*^−/−^ GC and with IL-21 affecting population dynamics—such as the rate of transition between LZ and DZ—rather than gene expression per se. Using the limma barcode approach for gene set enrichment analysis (see “Methods”), we compared these DE genes with an existing GC zone signature^42^, which revealed enrichment of DZ signature genes among transcripts up in WT vs *Il21r*^−/−^ and LZ signature enrichment among those up in *Il21r*^−/−^ vs WT (Fig. 5C). We compared also the DE gene list with those of the GC signatures of *Myc*, *FOXO1*-, and *mTOR*-^14,15,17^, and concluded that among these, the Myc signature strongly correlated with WT vs. *Il21r*^−/−^ (*p* = 1.9 × 10^−5^; Fig. 5D). Thus transcriptomic analysis of the impact of IL-21 on GC B cells was consistent with a distortion in zone representation and a deficiency in B cell proliferation.

## Discussion

While the importance of IL-21 in sustaining GC, and thus determining the quality and quantity of B cell memory, has been apparent for some time^31,32^, how this is achieved has been less clear. By quantifying GC zone development in conjunction with cell cycle phases during the first week of a TD response, we have determined that IL-21 functions to set the ratio of LZ to DZ cells and to promote the entry of antigen-specific LZ B cells into DNA synthesis. The necessity of this activity is revealed by the enlarged LZ and the accumulation of LZ B cells that remain in late G1 in *Il21r*^−/−^ GC. We have also mapped the distribution of the phases of the cell cycle within GC zones and determined gene expression differences in GC B cells arising from an absence of IL-21. Last, we determined that IL-21 can act directly on GC B cells to activate signaling pathways associated with proliferation, suggesting a basis for its role in the GC. Based on these and previous results, we propose that IL-21 promotes GC B cell proliferation to establish and then sustain normal GC zonal dynamics, and that in its absence, GC are destined for premature termination with a resultant insufficiency of affinity maturation and bone marrow PC.

We identified a deficit of LZ B cells entering into the S phase of the cell cycle in the absence of IL-21. Positive selection in the GC is mediated by Tfh-driven B cell proliferation whereby Tfh cell interaction with a LZ B cell induces transient expression of MYC, which is absolutely required for proliferation and development of the CB phenotype^13,14,43^. The amount of MYC expressed, while proportional to the subsequent proliferation^44,45^, may not be sufficient to sustain multiple rounds of proliferation of positively selected B cells in the DZ. It has been proposed that AP4, a MYC-dependent transcription factor, instead sustains proliferation in the DZ until it is diluted or degraded and falls below a threshold concentration required to maintain proliferation^20^. It has been proposed that IL-21 acts to sustain AP4 in DZ cells^20^. We suggest that IL-21 has a more complex, AP4-independent role in the GC, acting on LZ B cells to promote the transition from G1 to S phases. In support of this, we note that there is no distortion in zone representation in the absence of AP4 (ref. ^20^), yet this is a prominent feature of IL-21 deficiency. Also, IL-21 acting only through AP4 would not necessarily result in a decreased S phase of *Il21r*^−/−^ LZ B cells, as AP4 is downstream of MYC and operates after initiation of proliferation and mostly in the DZ^20^.

GC B cell proliferation still occurs in the absence of IL-21 (refs. ^31,32^), leading us to propose its action lowers the threshold of activation and thus increases the frequency of responding cells, analogous to that of IL2 in T cells^46^. In the case of IL-21, this effect appears to be mediated, at least to some extent, by PI3K signaling. IL-21 by itself is not mitogenic but our data indicate that by promoting AKT and S6 phosphorylation, IL-21R signaling influences the outcome of other signaling events such as those downstream of BCR stimulation in a way that shifts the outcome in favor of proliferation. In particular, BCR signaling is re-wired in GC B cells, resulting in limited AKT phosphorylation^40,47^, which may be partially compensated for by the presence of IL-21. In addition, STAT3, which is the dominant signal transduction mechanism of IL-21 in B cells^48,49^, is known to increase expression of a number of cell cycle-promoting genes including Cyclin D and cMyc^50–52^. While our RNA-seq analysis did not reveal differential expression between WT and *Il21r*^−/−^ GC B cells for these or many other cell cycle genes, this may reflect either a failure to reach significance due to experimental conditions or that pathways dependent on STAT3 are distinct from those described here. GC failure in the absence of STAT3 in B cells has been attributed to apoptosis^53^, while the defect we identify is in proliferation. It may be that in the absence of STAT3, cell survival is the dominant phenotype, while the effects of IL-21 deficiency reflect STAT3-independent components of IL-21R signaling. That is, in IL-21R- and IL-21-deficient mice, STAT3 may be activated in B cells by other cytokines, such as IL-6.

A striking property of *Il21r*^−/−^ GC is the representation of early G1 cells. In WT, most early G1 cells are in the DZ where the majority of cell cycling occurs^9,23^, whereas the bulk of *Il21r*^−/−^ early G1 cells have a LZ phenotype and are significantly over-represented in the LZ. That the *Il21r*^−/−^ LZ has a reduction in S phase cells suggests that IL-21 normally functions to promote the transition of LZ B cells from G1 to S/G2/M. But immediate return to the S phase is not the only fate available to LZ B cells—they may remain in G1, surviving by continued expression of pro-survival molecules^23,54^. The signals that determine survival of GC B cell in G1 are poorly understood, other than the shared requirement for continued expression of *Mcl1* (ref. ^55^). LZ B cells that lose affinity for antigen, contact with Tfh or FDC are lost from the GC. However, loss from the LZ is not a feature of IL-21 deficiency, indicating that its role is not cell survival per se but in cell behavior. This raises the question of which pathway *Il21r*^−/−^ early G1 B cells returning to the LZ take if the S phase is not available. While the dynamics of G1 cells in the LZ are unknown, our data indicate that IL-21 acts to limit the interval between re-acquisition of the LZ phenotype and re-entering cell division, thereby modulating the accumulation of such cells and the period for T cell-mediated selection and fate determination. That the number of G1 B cells the GC LZ can sustain can vary, raises interesting questions as to what mediates the persistence and turnover of B cells in the LZ.

The formation of GC zones, which are crucial to the efficient operation of affinity maturation, was described over 30 years ago^6,8,56^. However, insight into how the ratio of the two zones was established and then maintained has been lacking. We show here that IL-21 acts to suppress the appearance of CC phenotype cells, presumably by promoting division and thus CB phenotype cells that typify the expansion of antigen-specific B cells. This implies that IL-21 availability could influence zone distribution, and this could occur at the level of Tfh cell number and thus the amount of IL-21. An IL-21-reporter strain revealed approximately 30% of Tfh cells transcribed the *Il21* locus at any time^57^. Additionally, Tfh cell representation peaks early in the response and then remains constant for some time, making them—and presumably IL-21—a limiting resource in the GC^57–59^. This in turn may reduce either the amount of IL-21 per B cell or the number of B cells exposed to IL-21, which may influence the frequency of B cells entering the S phase, setting the zone representation and cell cycle distribution. Although our observations were in the absence of IL-21, it is attractive to speculate that IL-21 has a dose-dependent effect and indeed, dynamic regulation of IL-21 production by Tfh cells has been noted in GC in some responses^60^. Importantly, the effects of IL-21 we have noted appear to be unique as deficiency of IL-4, together with IL-21 a GC-critical cytokine^61^, does not induce a similar phenomenon.

The question of homogeneity of B cells within GC zones has only recently been explored^38^ in so far as addressing if LZ cells and DZ cells are homogeneous in the properties attributed to the zone. Our independent determination of the distribution of cell cycle phases across the spectrum of LZ and DZ phenotype cells confirms the clear relationship between phenotype and cell cycle activity^38^. We found that DNA synthesis in WT GC occurs most often in CXCR4^+^ DZ cells that are intermediate for CD86, while early G1 cells are most frequent in DZ cells with less CD86 than their S/G2/M precursors and late G1 cells are concentrated in LZ cells with the lowest amounts of CXCR4. This relationship of GC B cell phenotype with proliferation is consistent with the current model of multiple rounds of proliferation in the DZ for positively selected B cells^8,9^. The fate of DZ B cells expressing the lowest amounts of CD86, that remain in cell cycle but that do not show increases in size consistent with cell division, however, is an open question.

The finding that *Il21r*^−/−^ GC are numerically equal to WT early in a response but then collapse after 7–10 days suggests that their DZ, smaller both initially and with less ongoing input from the LZ, are ultimately unable to sustain the reaction. In this context, we saw no evidence for increased memory formation from *Il21r*^−/−^ GC, despite the increased fraction of cells in late G1 and the increased expression of nominal memory markers including *IL9r*^34^, *Sell*, and CD38. There are suggestions that “quiescence” in the LZ is a precursor for differentiation into memory, and while this may be the case, we would add that retention in G1 may be only one of several steps towards memory. Analyzing the population dynamics of LZ B cells relative to the phases of the cell cycle may reveal important processes underlying the generation of MBCs.

## Methods

### Mice, immunization and tissue recovery

All mutant mice were 8–12 generations backcrossed onto the C57Bl/6 background. IL-21 receptor deficient (*Il21r*^−/−^) were kindly provided by Dr. Warren Leonard, NHLBI, Bethesda MD; *Il21*^−/−^ by ZymoGenetics (Seattle, USA), and the FUCCI RG mice were constructed by crossing FUCCI Red (B6.B6D2-Tg(FUCCI)596Bsi) with FUCCI Green (B6.B6D2-Tg(FUCCI)504Bsi) mice, both obtained from the Riken BioResource Centre^33^. *Il21r*^−/−^ mice were crossed with FUCCI RG mice to generate both control (WT) and *Il21r*^−/−^ FUCCI report mice for bone marrow donation. All mice were bred and maintained in the specific pathogen-free (SPF) facilities of the Walter and Eliza Hall Institute of Medical Research (WEHI) or Monash Animal Research Platform (MARP). All experiments, except BrdU/EdU time course, used irradiated Ly5.1 mice (2 doses 5.5 Gy, minimum 2 h apart) that were reconstituted with either 2–5 × 10^6^ cells from *Il21r*^−/−^ or WT bone marrow cells. Chimeric reconstitutions were generated by using donor bone marrow cells mixed at a ratio of 1:1 from *Il21r*^−/−^ and Ly5.1 congenic strains (total cell number 2–5 × 10^6^), all bred and maintained at WEHI or MARP. Mice were allowed to reconstitute a minimum of 8 weeks before experimentation. Immunizations were with the hapten 4(hydroxy-3-nitrophenyl)acetyl coupled to the protein Keyhole Limpet Hemocyanin (NP-KLH) at a molar ratio of approximately 15–27:1. Antigen, 100 μg, was precipitated on alum and delivered by i.p. injection^62^. At the indicated times following immunization, mice were euthanized, spleens removed, and single-cell suspensions prepared for analysis with cells counted (CASY Cell Counter). The mice were housed at an ambient temperature of 20–22 °C, and they were kept under a 14 h day and 10 h night cycle. All procedures and experiments involving animals complied with all relevant regulations for animal research, and were conducted according to protocols approved by Animal Ethics Committee of either The Walter and Eliza Hall Institute or the Alfred Research Alliance.

### Antibodies and flow cytometry

Antibodies for flow cytometry used in this work were purified and conjugated in-house unless otherwise indicated and were tested to ensure the optimal concentration was used. B cell antibody cocktail panels contained combinations of the following: IgM-Biotin (clone 331.12; 1 in 200 dilution), Gr1-Biotin (clone Rb6-8C5; 1 in 200 dilution), StAv-BUV737 (BD Biosciences, Cat. 564293; 1 in 200 dilution), IgM-BV510 (clone R6-60.2, BD Biosciences, Cat. 563118; 1 in 200 dilution), IgD-BV711 (clone 11-26c.2a; BD Biosciences, Cat. 564275; 1 in 200 dilution), IgG1-APC (clone X56, BD Biosciences, Cat. 550874; 1 in 200 dilution), CD19-BUV395 (clone 1D3, BD Biosciences, Cat. 563557; 1 in 200 dilution), CD19-BUV737 (clone 1D3, BD Biosciences, Cat. 564296; 1 in 200 dilution), CD138-BV650 (clone 281.2, BD Biosciences, Cat. 564068; 1 in 200 dilution), CD86-BV605 (clone GL1, BD Biosciences, Cat. 563055; 1 in 200 dilution), CXCR4-BV421 (clone 2B11, BD Biosciences, Cat. 562738; 1 in 50 dilution), Fas-PECy7 (clone Jo2, BD Biosciences, Cat. 557653; 1 in 200 dilution), Fas-BUV395 (clone Jo2, BD Biosciences, Cat. 740254; 1 in 200 dilution), CD38-A680 (clone NIMR5; 1 in 200 dilution), NP-APC (NIP-CAP-OSu, Biosearch Technologies, Cat. N-1110-100; 1 in 200 dilution), NP-PE (NIP-CAP-OSu, Biosearch Technologies Cat. N-1110-100 and Thermo Fisher Scientific, Cat. P801; 1 in 200 dilution). Prior to staining with B cell antibody cocktail, splenocytes were treated to remove cytophilic antibodies with an acid wash as follows: 10× acid wash solution (0.85 M NaCl, 0.05 M KCl, 0.5 M Na acetate, pH 4.0) was diluted in dH_2_O containing 1% fetal calf serum (FCS) and chilled on ice. Cells were pelleted in a 10 mL tube by centrifugation and resuspended in 100 μL 1× acid wash solution for 1 min on ice and subsequently neutralized by filling tube with phosphate-buffered saline (PBS) containing 2% FCS. T cell antibody cocktail panels contained combinations of the following: CXCR5-Biotin (clone 2G8, BD Biosciences, Cat. 551960; 1 in 50 dilution), StAv-BV650 (clone BD 5, Biosciences, Cat. 63855; 1 in 200 dilution), PD1-PECy7 (clone RMP-30, Biolegend, Cat. 109110; 1 in 200 dilution), CD62L-PB/V450 (clone MEL-14, BD Biosciences, Cat. 560507; 1 in 200 dilution), CD44-APC (clone IM7, BD BrdU, BD Biosciences, Cat. 559250; 1 in 200 dilution), CD4-A680 (clone GK1.5; 1 in 200 dilution), CD19-APC (clone 1D3, BD Biosciences, Cat. 550992; 1 in 200 dilution), CD19-PE (clone 1D3, eBioscience, Cat. 12-0193-83; 1 in 200 dilution). For chimeric mice studies the following congenic markers were used: Ly5.1-PECy7 (clone A20, eBioscience, Cat. 25-0453-82; 1 in 200 dilution), Ly5.2-FITC (clone 104, BD Biosciences, Cat. 553772; 1 in 200 dilution), Ly5.1-PerCPCy5.5 (clone A20, eBioscience, Cat. 45-0453-80; 1 in 200 dilution). All flow cytometry staining was performed with 5–10 × 10^6^ splenocytes and performed in PBS containing 2% FCS, 1% normal rat serum (NRS), anti-CD16/32 (clone 2.4G2, in-house hybridoma; 1 in 50 dilution). Samples were acquired on a BD Fortessa x20 with BD FACS DIVA software, in PBS containing FCS and viability dye, either SYTOX Blue (Thermo Fisher Scientific, Cat. S34857) or FluoroGold (Santa Cruz Biotech, Cat. SC-358883). For Ki67 staining, cells were fixed using eBioscience Foxp3/Transcription Factor Staining Buffer Set (Thermo Fisher Cat. 00-5523-00) according to the manufacturer’s instructions. Ki-67-PE (Clone 11F6, BioLegend cat. #151209; 1 in 100 dilution). Flow cytometry data were analyzed using FlowJo versions 9.9.6 and 10 and statistical analysis performed using GraphPad Prism 7 and 8.

### EdU and BrdU DNA incorporation

*Il21*^−/−^ or WT mice were immunized as above with NP-KLH in alum. On day 6 post immunization, mice were injected i.p. with 100 µL of 10 mg/mL (5-ethynyl-2′-deoxyuridine) (EdU, Invitrogen, Cat. A10044) in PBS and 17 h later, injected i.p. with 200 µL of 10 mg/mL 5-bromo-2′-deoxyuridine (BrdU, BD Biosciences, Cat. 559619) in PBS. One hour later, mice were killed by cervical dislocation, spleens harvested, and single-cell suspensions made. Following red blood cell lysis and determination of cell numbers (Coulter counter), 2 × 10^7^ cells were suspended in PBS containing 2% FCS, 1% NRS, anti-CD16/32 (clone 2.4G2, in-house hybridoma; 1 in 50 dilution), and antibodies to CD138-BV650 (Clone 281.2, BD Biosciences, Cat. 564068; 1 in 200 dilution), IgD-Alexa Fluor 680 (clone 11-26c.2a, in-house hybridoma and conjugated using Alexa Fluor™ 680 NHS Ester, Thermo Fischer Scientific, Cat. A20008; 1 in 200 dilution), CD38-BV786 (clone Ab90, BD Biosciences, Cat. 740887; 1 in 200 dilution), IgG1-APC (clone X56, BD Biosciences, Cat. 550874; 1 in 200 dilution), CD95-BUV395 (clone Jo2, BD Biosciences, Cat. 740254; 1 in 200 dilution), CD19-BUV737 (clone ID3, BD Biosciences, Cat. 564296; 1 in 200 dilution), CXCR4-BV711 (clone 2B11, BD Biosciences, Cat. 740734; 1 in 50 dilution). Following 30 min incubation on ice and washing with PBS/2% FCS, cells were suspended in 200 µL BD cytofix/cytoperm (BD Biosciences, Cat. 554722) and incubated for 15 min on ice. After washing with 2 mL BD Perm/Wash (BD Biosciences, Cat. 554723), cells were suspended in 150 µL permeabilization buffer plus (BD Biosciences, Cat. 561651) on ice for 10 min followed by 2 mL Perm/Wash, 5 min fixation with 150 µL Cytofix/Cytoperm, and another wash with 2 mL Perm/Wash. For DNA digestion, cell pellets were suspended in a mixture of 30 µL DNAse 1 stock (Sigma, Cat. D4513-1VL, 1 mg/mL in ddH_2_O) with 70 µL PBS and incubated at 37 °C for 1 h. After washing with 2 mL Perm/Wash, EdU Click-iT reaction mixture (Click-iT Plus EdU Pacific Blue Flow Cytometry Assay Kit, Thermo Fisher, Cat. C10636) was prepared by mixing 87.5 µL PBS with 2 µL copper protectant, 0.5 µL fluorescent azide and 100 µL 1× reaction buffer additive (plus) per sample. Cells were resuspended in 200 µL Click-iT reaction mixture and incubated for 20 min at room temperature. After washing with 2 mL Perm/Wash, cells were resuspended in Perm/Wash containing anti-CD16/32 (1 in 50 dilution), NP-conjugated PE (in-house conjugated; 1 in 200 dilution), anti-CD86-PE-Cy7 (clone GL1, BD Biosciences, Cat. 560582; 1 in 200 dilution), and anti-BrdU-FITC (FITC BrdU Flow Kit, BD Biosciences, Cat. 559619; 1 in 200 dilution) for 30 min at room temperature. After washing with 2 mL Perm/Wash, pellets were resuspended in PBS and acquired using BD Fortessa X20 with BD FACS DIVA software. Flow cytometry data were analyzed using FlowJo 10 and statistical analysis performed using GraphPad Prism 7 and 8.

### Cell sorting, RNA sequencing, and data analysis

NP-specific IgG1^+^ GC B cells were identified and sorted from the spleens of *Il21r*^−/−^ or WT mice day 7, 10, and 14 after immunization. A total of 9–10 mice were used per genotype from two independent immunizations. IgG1^+^ cells were enriched using magnetic beads coated with anti-mouse IgG1 (Miltenyi Biotec, Cat. 130-047-101; 1 in 200 dilution). MACS enrichment was performed as per the manufacturer instructions using MACS LS columns (Miltenyi Biotec, Cat. 130-042-401) except no primary antibody was used and cells were resuspended in 90 μL of buffer per 10^7^ cells with 10 μL of anti-IgG1 microbeads subsequently added per 10^7^ cells. Following enrichment and staining (CD138-5(6)CF (clone 281; in-house generated; 1 in 200 dilution), IgM-5(6)CF (clone 331.12; in-house generated; 1 in 200 dilution), IgD-5(6)CF (clone 11-26C; in-house generated; 1 in 200 dilution), Gr1-5(6)CF (clone Rb6-8C5; in-house generated; 1 in 200 dilution), NP-PE (NIP-CAP-OSu, Biosearch Technologies Cat. N-1110-100 and Thermo Fisher Scientific, Cat. P801; in-house conjugated; 1 in 200 dilution), IgG1-APC (clone X56, BD Biosciences, Cat. 550874; 1 in 200 dilution), CD38-A680 (clone NIMR5, in-house generated; 1 in 200 dilution), B220-PB (clone RA3-6B2, BD Biosciences, Cat. 558108; 1 in 200 dilution)), antigen-specific GC B cells (CD138^−^IgM^−^IgD^−^Gr1^−^B220^+^NP^+^IgG1^+^CD38^−^) were sorted on a BD FACSAria II Cell Sorter. FACS staining was performed in PBS containing 2% FCS, 1% normal rat serum (NRS), anti-CD16/32 (clone 2.4G2, in-house hybridoma; 1 in 50 dilution), and viable cells identified as negative for propidium iodide (Millipore, Cat. 537059). RNA was isolated from cells using Qiagen RNeasy Plus Micro Kits (Cat No 74034), as per the manufacturer instructions. RNA-seq libraries were prepared by the Australian Genome Research Facility (Melbourne, Australia) using the TruSeq RNA Library Prep Kit v2. Libraries were pooled and clustered with the Illumina cBot system using TruSeq SR Cluster Kit v3 reagents followed by sequencing on the Illumina CASAVA pipeline 1.8.2 HiSeq 2000 system with TruSeq SBS Kit v3 reagents with 101 cycles. RNA-seq was performed with 100 bp single end reads to an approximate depth of 30 million reads after pooling technical replicates. Reads were aligned to the mouse reference genome mm10 using Rsubread^63^. Read counts were summarized at the gene level using featureCounts and Rsubread’s inbuilt RefSeq annotation^64^. Differential expression analysis was conducted using the edgeR package (version 4.0.34)^65,66^. Genes with very low counts (as judged by negative values from edgeR’s aveLogCPM function) were excluded from downstream analysis. Y-chromosome genes were filtered, as well as Xist and genes corresponding to the Ig hypervariable regions (i.e. Ig “something” v), leaving 11,130 genes. Library sizes were normalized using the trimmed mean of *M*-values (TMM) method. Genewise negative binomial dispersion parameters were estimated by estimateDisp with robust=TRUE and an additive model with a linear effect for day since immunization. Generalized linear models were then refitted with separate *Il21r*^−/−^ vs WT effects for each day and differential expression was assessed using likelihood ratio tests. Gene set enrichment tests for LZ and DZ signatures were performed using the camera function^67^ of the limma package (version 3.1.6.8)^68^. Barcode enrichment plot was drawn using limma’s barcodeplot function. A gene signature for light vs dark zone GC B cells was obtained from Table S1 of Victora et al. (2010) using genes with expression changes of twofold or greater. A Myc-deficiency signature was obtained from Table S2 of ref. ^14^; FOXO1-deficiency signature was obtained by reanalysing microarray data from ref. ^17^; and mTOR-deficiency signature from NCBI GEO GSE98778 (ref. ^15^). Normalized log_2_-expression values were downloaded from GEO series GSE68043 and analyzed using the limma package^68^. A contrast was formed between the FOXO1 KO group and the average of the DZ and LZ control groups. Differential expression was assessed by moderated *t*-tests with trended empirical Bayes^69^ resulting in 83 upregulated and 162 downregulated genes in the FOXO1 samples (FDR < 0.05).

### Feeder cell preparation, B cell isolation, and iGC B cell culture

For B cell cultures, feeder cells expressing CD40L and secreting BAFF^39,70^ were seeded at a density of 1.5 × 10^5^ or 6 × 10^5^ cells per well in 24- or 6-well plates, respectively (Corning Inc., Corning, NY, USA). Spleen, single-cell suspensions were labeled with Cell Trace Violet (CTV, Molecular Probes, Cat. C34557) and B cells isolated by MACS negative enrichment (B cell isolation kit, Miltenyi Biotec, Cat. 130-090-862) according to the manufacturer’s instructions. Purified cells were counted by a hemocytometer and 2.0 × 10^4^ or 1.6 × 10^5^ B cells per well were seeded in a 24- or 6‐well plate in 1.3 or 6.5 mL of media, respectively. Media contained RPMI-1640 (Gibco, Cat. 11875119) supplemented with 10% FCS, 1 mM sodium pyruvate, 55 μM 2-mercaptoethanol, 100 U/mL–100 μg/mL penicillin–streptomycin^39^. The cells were cultured at 37 °C with 5% CO_2_. On day 2 of culture, where indicated, recombinant mouse IL-21 (R&D Systems) was added for 2 h to a final concentration of 10 ng/mL. Subsequently cells were harvested, washed, and either analyzed by flow cytometry or re-seeded on fresh feeders in the presence of anti-CD40L (clone MR1; a gift from R. Noelle^71^) antibody at 10 μg/mL to block CD40L stimulation. All culture conditions were in duplicate or triplicate for each condition at each harvest time point, days 2 and 3. For flow cytometry analysis, prior to harvest, counting beads were added and a single well per condition was stained with the following antibody cocktail in PBS containing 2% FCS: CD19-APC (clone 1D3, BD Biosciences, Cat. 550992; 1 in 200 dilution) or CD19-PECy7 (clone 1D3, eBioscience, Cat. 25-0193-82; 1 in 200 dilution), IgD-PE (clone 1126.2a, Southern Biotec, Cat. 1120-09; 1 in 200 dilution) or IgD-A680 (clone 1126.2a, in-house; 1 in 200 dilution), CD138-BV650 (clone 281.2, BD Biosciences, Cat. 564068; 1 in 200 dilution) or CD138-APC (clone 281.2, BD Biosciences, Cat. 561705; 1 in 200 dilution). Cells were acquired on a BD LSR Fortessa with BD FACS DIVA software and viable cells identified as negative for propidium iodide (Millipore, Cat. 537059) or Fixable Viability Dye eFluor780 (eBioscience, Cat. 65-0865-14). Analyses were performed with FlowJo version 9.9.6.

### Histology and confocal microscopy

Spleens were fixed in 4% methanol-free formaldehyde (Sigma, Cat. 28906) in PBS at 4 °C for 3.5 h followed by overnight incubation in 30% sucrose in PBS and freezing in OCT (Tissue-Tec, Cat. 4583). Spleens were cut into 10 µM sections using a Leica CM1850 cryostat and the tissue was transferred onto microscope slides. The tissue was blocked with PBS + 5% FCS + 2% rat serum + anti-FcR (clone 2.4G2, in-house hybridoma) for 1 h followed by staining with antibodies to Bcl6-CF568 (clone 7D1-10, in-house purified and conjugated to CF568 (Biotium, Cat. 92215); 1 in 200 dilution), CD35-biotin (BD Biosciences, Cat. 553816; 1 in 200 dilution), CD45.1-eFluor450 (clone A20, eBioscience, Cat. 48-0453-82; 1 in 200 dilution), CD45.2-FITC (clone 104, BD Biosciences, Cat. 553772; 1 in 200 dilution), and B220-AF647 (clone RA3-6B2, in-house purified and conjugated to Alexa fluor 647; 1 in 200 dilution) in a humidified chamber at 4 °C overnight. Slides were washed and stained with Streptavidin-Pacific orange (Thermo Fischer, Cat. S32365) at room temperature for 1 h. After washing twice for 1 h with PBS, slides were mounted using DAKO fluorescent mounting medium (DAKO, Cat. S3023) and Menzel X1000 Coverslip #1.5. Images were acquired on a Nikon AR1 confocal microscope with a CFI Plan Fluor ×20 MI lens using glycerol as immersion medium.

### GC B cell phosphoflow analysis

Splenocytes from C57Bl/6 mice were isolated and the cells of two spleens pooled per sample. GC B cell was enriched by magnetic sorting by staining with biotinylated antibodies to GR1 (clone 8C5, WEHI Antibody Facility; 1 in 200 dilution), IgD (clone 11-26, Southern Biotech, Cat. 1120-08; 1 in 200 dilution), CD4 (clone GK1.5, WEHI Antibody Facility; 1 in 200 dilution), CD8 (clone YTS.169, WEHI Antibody Facility; 1 in 200 dilution), and CD138 (clone 281-2, BD Biosciences, Cat. 553713; 1 in 200 dilution) followed by staining with anti-biotin microbeads (Miltenyi Biotec, Cat. 130-090-485; 1 in 200 dilution) and depletion using LS Columns (Miltenyi Biotec, Cat. 130-042-401) supported by a MACS separator magnetic stand (Miltenyi Biotec). The flow through containing GC B cells was collected for stimulation. Per experimental condition, 1.5 × 10^6^ cells were used. Enriched GC B cells were either left unstimulated or stimulated with IL-21 (20 ng/mL; PeproTech, Cat. 210-21-100) or anti-CD40 (20 ng/mL; clone IC10, WEHI Antibody Facility) for 2 h. For BCR stimulation, anti-Igκ and anti-Igλ cells were incubated with biotinylated rat anti-Igκ (100 ng/mL; clone 187.1, WEHI Antibody Facility) and anti-Igλ (100 ng/mL; clone JC5, WEHI Antibody Facility) during the last 15 min of the 2 h incubation, followed by the addition of avidin (10 µg/mL) during the last minute. Stimulation was stopped by fixation, followed by permeabilization with the BD phosphoflow staining reagents as per the manufacturer’s instructions (BD Biosciences). Cells were stained for flow cytometry with anti-p-S6 (Ser235/236)-PE-Cy7 (clone D57.2.2E, Cell Signaling, Cat. 34411S; 1 in 100 dilution), anti-pAKT (S473)-PE (clone M89-61, BD Biosciences, Cat. 561671; 1 in 40 dilution), PNA-FITC (Vector Laboratories, Cat. FL-1071; 1 in 200 dilution), B220-BV421 (clone RA3-B62, BD Biosciences, Cat. 562922; 1 in 200 dilution), IgD-BV711 (clone 11-26c.2a, BD Biosciences, Cat. 564275; 1 in 200 dilution), CD4-PerCP-Cy5.5 (clone RM4-5, BD Biosciences, Cat. 550954; 1 in 200 dilution). Flow cytometry was performed on a 5-laser LSRFortessa X20 flow cytometer (BD Biosciences) and collected with BD FACS DIVA software. GC B cells were identified as being B220^+^CD4^−^IgD^−^PNA^+^. Flow cytometry data were analyzed with FlowJo 10 software (BD).

### Reporting summary

Further information on research design is available in the Nature Research Reporting Summary linked to this article.

## Supplementary information

Supplementary Information

Reporting Summary

## Data Availability

The RNA-seq data generated in this study have been deposited in the GEO database under accession code GSE184475. The microarray data used in this study are available in the NCBI Gene Expression Omnibus under accession codes GSE98778 and GSE68043. All other data are available in the article and its Supplementary files or from the corresponding author upon reasonable request. Source data are provided with this paper.

## Source data

Source Data

## References

[1] Cyster JG, Allen CDC (2019). B cell responses: cell interaction dynamics and decisions. Cell.

[2] Mesin L, Ersching J, Victora GD (2016). Germinal center B cell dynamics. Immunity.

[3] Tarlinton D, Good-Jacobson K (2013). Diversity among memory B cells: origin, consequences, and utility. Science.

[4] Weisel F, Shlomchik M (2017). Memory B cells of mice and humans. Annu. Rev. Immunol..

[5] Bachmann MF, Odermatt B, Hengartner H, Zinkernagel RM (1996). Induction of long-lived germinal centers associated with persisting antigen after viral infection. J. Exp. Med..

[6] MacLennan IC (1994). Germinal centers. Annu. Rev. Immunol..

[7] Ridderstad A, Tarlinton DM (1998). Kinetics of establishing the memory B cell population as revealed by CD38 expression. J. Immunol..

[8] Victora GD, Nussenzweig MC (2012). Germinal centers. Annu. Rev. Immunol..

[9] Gitlin AD, Shulman Z, Nussenzweig MC (2014). Clonal selection in the germinal centre by regulated proliferation and hypermutation. Nature.

[10] Gitlin AD (2015). Humoral immunity. T cell help controls the speed of the cell cycle in germinal center B cells. Science.

[11] Crotty S (2014). T follicular helper cell differentiation, function, and roles in disease. Immunity.

[12] Shulman Z (2014). Dynamic signaling by T follicular helper cells during germinal center B cell selection. Science.

[13] Calado DP (2012). The cell-cycle regulator c-Myc is essential for the formation and maintenance of germinal centers. Nat. Immunol..

[14] Dominguez-Sola D (2012). The proto-oncogene MYC is required for selection in the germinal center and cyclic reentry. Nat. Immunol..

[15] Ersching J (2017). Germinal center selection and affinity maturation require dynamic regulation of mTORC1 kinase. Immunity.

[16] Dominguez-Sola D (2015). The FOXO1 transcription factor instructs the germinal center dark zone program. Immunity.

[17] Sander S (2015). PI3 kinase and FOXO1 transcription factor activity differentially control B cells in the germinal center light and dark zones. Immunity.

[18] Allen CD (2004). Germinal center dark and light zone organization is mediated by CXCR4 and CXCR5. Nat. Immunol..

[19] Allen CD, Okada T, Cyster JG (2007). Germinal-center organization and cellular dynamics. Immunity.

[20] Chou C (2016). The transcription factor AP4 mediates resolution of chronic viral infection through amplification of germinal center B cell responses. Immunity.

[21] Inoue T (2017). The transcription factor Foxo1 controls germinal center B cell proliferation in response to T cell help. J. Exp. Med..

[22] Bannard O (2013). Germinal center centroblasts transition to a centrocyte phenotype according to a timed program and depend on the dark zone for effective selection. Immunity.

[23] Stewart I, Radtke D, Phillips B, McGowan SJ, Bannard O (2018). Germinal center B cells replace their antigen receptors in dark zones and fail light zone entry when immunoglobulin gene mutations are damaging. Immunity.

[24] Mayer, C. T. et al. The microanatomic segregation of selection by apoptosis in the germinal center. *Science* **358**, eaao2602 (2017). 10.1126/science.aao2602 PMC5957278 28935768

[25] Kepler TB, Perelson AS (1993). Cyclic re-entry of germinal center B cells and the efficiency of affinity maturation. Immunol. Today.

[26] Collins CM, Speck SH (2015). Interleukin 21 signaling in B cells is required for efficient establishment of murine gammaherpesvirus latency. PLoS Pathog..

[27] Perez-Mazliah D (2015). Disruption of IL-21 signaling affects T cell-B cell interactions and abrogates protective humoral immunity to malaria. PLoS Pathog..

[28] Tangye SG, Ma CS, Brink R, Deenick EK (2013). The good, the bad and the ugly—TFH cells in human health and disease. Nat. Rev. Immunol..

[29] Zotos D, Tarlinton DM (2012). Determining germinal centre B cell fate. Trends Immunol..

[30] Bessa J, Kopf M, Bachmann MF (2010). Cutting edge: IL-21 and TLR signaling regulate germinal center responses in a B cell-intrinsic manner. J. Immunol..

[31] Linterman MA (2010). IL-21 acts directly on B cells to regulate Bcl-6 expression and germinal center responses. J. Exp. Med..

[32] Zotos D (2010). IL-21 regulates germinal center B cell differentiation and proliferation through a B cell-intrinsic mechanism. J. Exp. Med..

[33] Sakaue-Sawano A (2008). Visualizing spatiotemporal dynamics of multicellular cell-cycle progression. Cell.

[34] Wang Y (2017). Germinal-center development of memory B cells driven by IL-9 from follicular helper T cells. Nat. Immunol..

[35] Dowling MR (2014). Stretched cell cycle model for proliferating lymphocytes. Proc. Natl Acad. Sci. USA.

[36] Jandl C (2017). IL-21 restricts T follicular regulatory T cell proliferation through Bcl-6 mediated inhibition of responsiveness to IL-2. Nat. Commun..

[37] Victora GD (2012). Identification of human germinal center light and dark zone cells and their relationship to human B-cell lymphomas. Blood.

[38] Kennedy DE (2020). Novel specialized cell state and spatial compartments within the germinal center. Nat. Immunol..

[39] Nojima T (2011). In-vitro derived germinal centre B cells differentially generate memory B or plasma cells in vivo. Nat. Commun..

[40] Luo W, Weisel F, Shlomchik MJB (2018). Cell receptor and CD40 signaling are rewired for synergistic induction of the c-Myc transcription factor in germinal center B cells. Immunity.

[41] Zeng R (2007). The molecular basis of IL-21-mediated proliferation. Blood.

[42] Victora GD (2010). Germinal center dynamics revealed by multiphoton microscopy with a photoactivatable fluorescent reporter. Cell.

[43] Ise W (2018). T follicular helper cell-germinal center B cell interaction strength regulates entry into plasma cell or recycling germinal center cell fate. Immunity.

[44] Heinzel S (2017). A Myc-dependent division timer complements a cell-death timer to regulate T cell and B cell responses. Nat. Immunol..

[45] Finkin S, Hartweger H, Oliveira TY, Kara EE, Nussenzweig MC (2019). Protein amounts of the MYC transcription factor determine germinal center B cell division capacity. Immunity.

[46] Deenick EK, Gett AV, Hodgkin PD (2003). Stochastic model of T cell proliferation: a calculus revealing IL-2 regulation of precursor frequencies, cell cycle time, and survival. J. Immunol..

[47] Khalil AM, Cambier JC, Shlomchik MJ (2012). B cell receptor signal transduction in the GC is short-circuited by high phosphatase activity. Science.

[48] Leonard W. J. & Wan C. K. IL-21 signaling in immunity. *F1000Res* **5**, F1000 Faculty Rev-224 (2016). 10.12688/f1000research.7634.1 PMC4770986 26966515

[49] Tangye, S. G. & Ma, C. S. Regulation of the germinal center and humoral immunity by interleukin-21. *J. Exp. Med*. **217**, e20191638 (2020). 10.1084/jem.20191638 PMC7037251 31821441

[50] Banerjee K, Resat H (2016). Constitutive activation of STAT3 in breast cancer cells: a review. Int. J. Cancer.

[51] Bromberg JF (1999). Stat3 as an oncogene. Cell.

[52] Leslie K (2006). Cyclin D1 is transcriptionally regulated by and required for transformation by activated signal transducer and activator of transcription 3. Cancer Res..

[53] Kane A, Lau A, Brink R, Tangye SG, Deenick EK (2016). B-cell-specific STAT3 deficiency: insight into the molecular basis of autosomal-dominant hyper-IgE syndrome. J. Allergy Clin. Immunol..

[54] Carrington EM (2010). BH3 mimetics antagonizing restricted prosurvival Bcl-2 proteins represent another class of selective immune modulatory drugs. Proc. Natl Acad. Sci. USA.

[55] Vikstrom I (2010). Mcl-1 is essential for germinal center formation and B cell memory. Science.

[56] Camacho SA, Kosco-Vilbois MH, Berek C (1998). The dynamic structure of the germinal center. Immunol. Today.

[57] Luthje K (2012). The development and fate of follicular helper T cells defined by an IL-21 reporter mouse. Nat. Immunol..

[58] Deenick EK (2010). Follicular helper T cell differentiation requires continuous antigen presentation that is independent of unique B cell signaling. Immunity.

[59] Song W, Craft J (2019). T follicular helper cell heterogeneity: Time, space, and function. Immunol. Rev..

[60] Weinstein JS (2016). TFH cells progressively differentiate to regulate the germinal center response. Nat. Immunol..

[61] Ozaki K (2002). A critical role for IL-21 in regulating immunoglobulin production. Science.

[62] Blink EJ (2005). Early appearance of germinal center-derived memory B cells and plasma cells in blood after primary immunization. J. Exp. Med.

[63] Liao Y, Smyth GK, Shi W (2019). The R package Rsubread is easier, faster, cheaper and better for alignment and quantification of RNA sequencing reads. Nucleic Acids Res..

[64] Liao Y, Smyth GK, Shi W (2014). featureCounts: an efficient general purpose program for assigning sequence reads to genomic features. Bioinformatics.

[65] McCarthy DJ, Chen Y, Smyth GK (2012). Differential expression analysis of multifactor RNA-Seq experiments with respect to biological variation. Nucleic Acids Res..

[66] Robinson MD, McCarthy DJ, Smyth GK (2010). edgeR: a Bioconductor package for differential expression analysis of digital gene expression data. Bioinformatics.

[67] Wu D, Smyth GK (2012). Camera: a competitive gene set test accounting for inter-gene correlation. Nucleic Acids Res..

[68] Ritchie ME (2015). limma powers differential expression analyses for RNA-sequencing and microarray studies. Nucleic Acids Res..

[69] Phipson B, Lee S, Majewski IJ, Alexander WS, Smyth GK (2016). Robust hyperparameter estimation protects against hypervariable genes and improves power to detect differential expression. Ann. Appl Stat..

[70] Robinson MJ (2019). BAFF, IL-4 and IL-21 separably program germinal center-like phenotype acquisition, BCL6 expression, proliferation and survival of CD40L-activated B cells in vitro. Immunol. Cell Biol..

[71] Noelle RJ (1992). A 39-kDa protein on activated helper T cells binds CD40 and transduces the signal for cognate activation of B cells. Proc. Natl Acad. Sci. USA.

