# Supplementary material for: The concerted change in the distribution of cell cycle phases and zone composition in germinal centers is regulated by IL-21
Source: Nat Commun. 2021 Dec 9;12:7160. doi: 10.1038/s41467-021-27477-0 (PMC8660905; doi:10.1038/s41467-021-27477-0)
Supplement: Supplementary file 1 — Supplementary Information [file 41467_2021_27477_MOESM1_ESM.pdf]

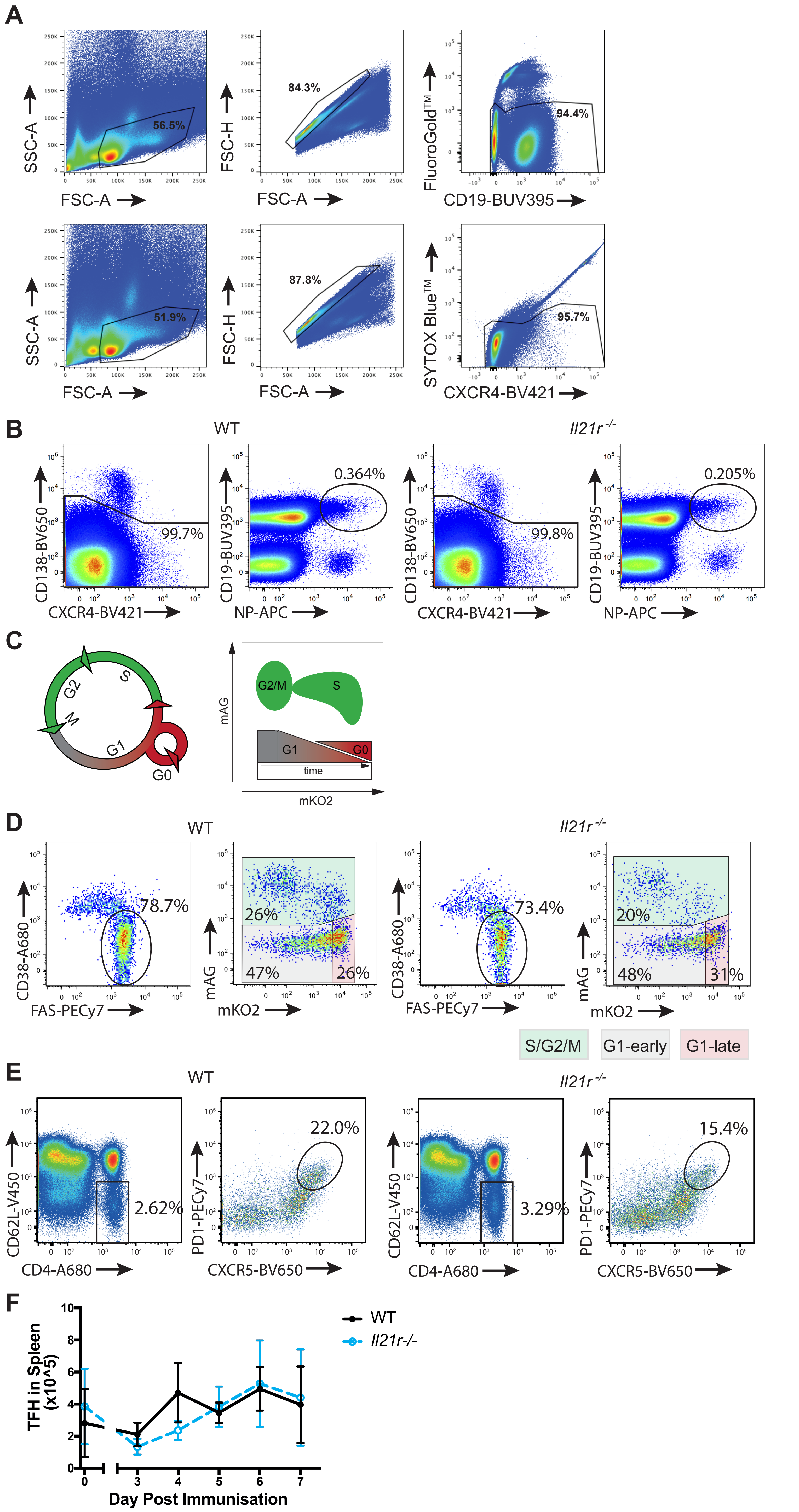


**Supplementary Figure 1. Identification of antigen-specific B cells with cell cycle stages and Tfh cells.** (A) Representative sequential flow cytometry electronic gates used for analysis of viable lymphocytes from the spleen. Either the top or lower panel gating strategy was used for all data shown in Figures 1, 2 and 3, and Supplementary Figures 1, and 5. (B) Representative flow cytometry gating from d7 NP-KLH in alum immunized FUCCI WT (left) and FUCCI *Il21r-/-* (right) mice for NP-binding B cells (CD138-CD19+NP+) amongst total splenic lymphocytes. (C) Schematic representation of cell cycle phase dependent monomeric Azami Green (mAG, green) and monomeric Kusabira Orange (mKO2, red) expression in FUCCI mice (left) and schematic depiction of their fluorescence as detected by flow cytometry (right). mKO2 expression intensity depends on time spent in G1/G0. (D) Representative flow cytometry gating for GC B cells (Fas+CD38-; left graph) for each genotype amongst NP-binding B cells shown in (B). Representative splenic FUCCI, mKO2 and mAG, expression profiles of NP-binding GC cells (right graph) for each genotype. Green (mAG+), red (mKO2hi) and grey (mAG- mKO2lo/-) boxes indicate electronic gates used to identify cells in S/G2/M, G1-early and G1-late, respectively. (E) Representative flow cytometry gating from NP-KLH in alum immunized WT (upper) and *Il21r-/-* (lower) mice for splenic T follicular helper (Tfh) cells (CD4+CD62L-PD1+CXCR5+). (F) Total number of Tfh cells in the spleen as gated in (E) for unimmunized and immunized mice of the indicated genotypes at times indicated. Data presented as mean ± SD from two independent experiments with 3-8 mice per time point.

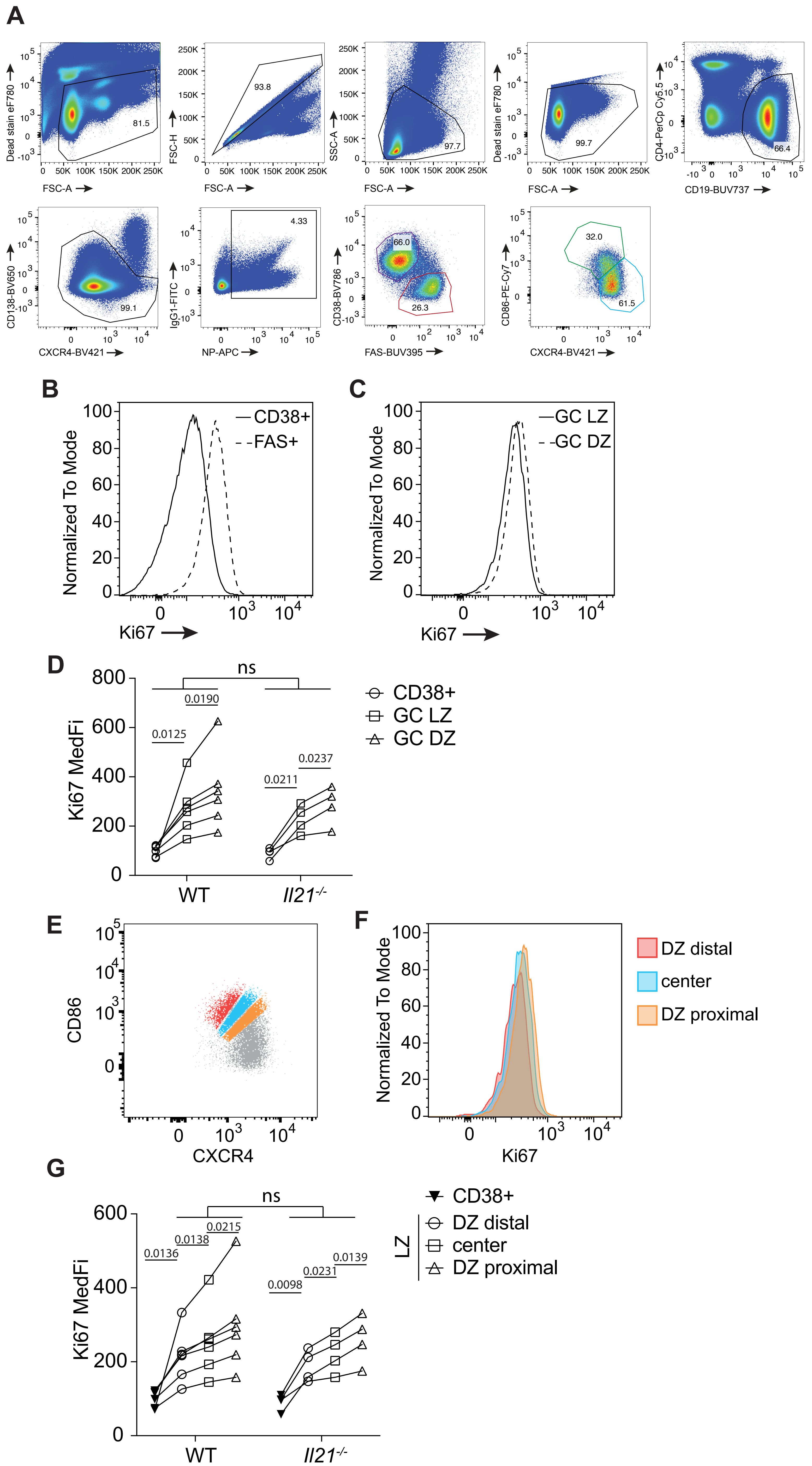

**Supplementary Figure 2. GC LZ B cells remain in G1 in the absence of IL-21 signalling, and express WT amounts of Ki67.** (A) Sequential gating strategy to identify NP binding CD38+ (purple) and FAS+ B cells (red) among splenocytes from WT and *Il21-/-* mice, day 7 post ip immunization with NP-KLH in alum. FAS+ B cells were further devided into GC DZ (blue) and LZ (green). These electronic gates were applied to the following figure analyses. Exemplary Ki67 staining of (B) NP+CD38+ or NP+FAS+ B cells, (C) within GC zones and (D) statistical analysis. Exemplary staining of (E) GC zones and LZ sub-fractions (distal, center, proximal as red, blue, orange respectively) with (F) derived Ki67 staining and (G) statistical analysis. Statistical analyses were performed using paired (within WT or *Il21-/-* mice) or unpaired t test (between genotypes). Exact p-values are shown with those ≤ 0.05 considered significant. Data are from one experiment, n=6 and n=4 for WT and Il21-/- respectively.

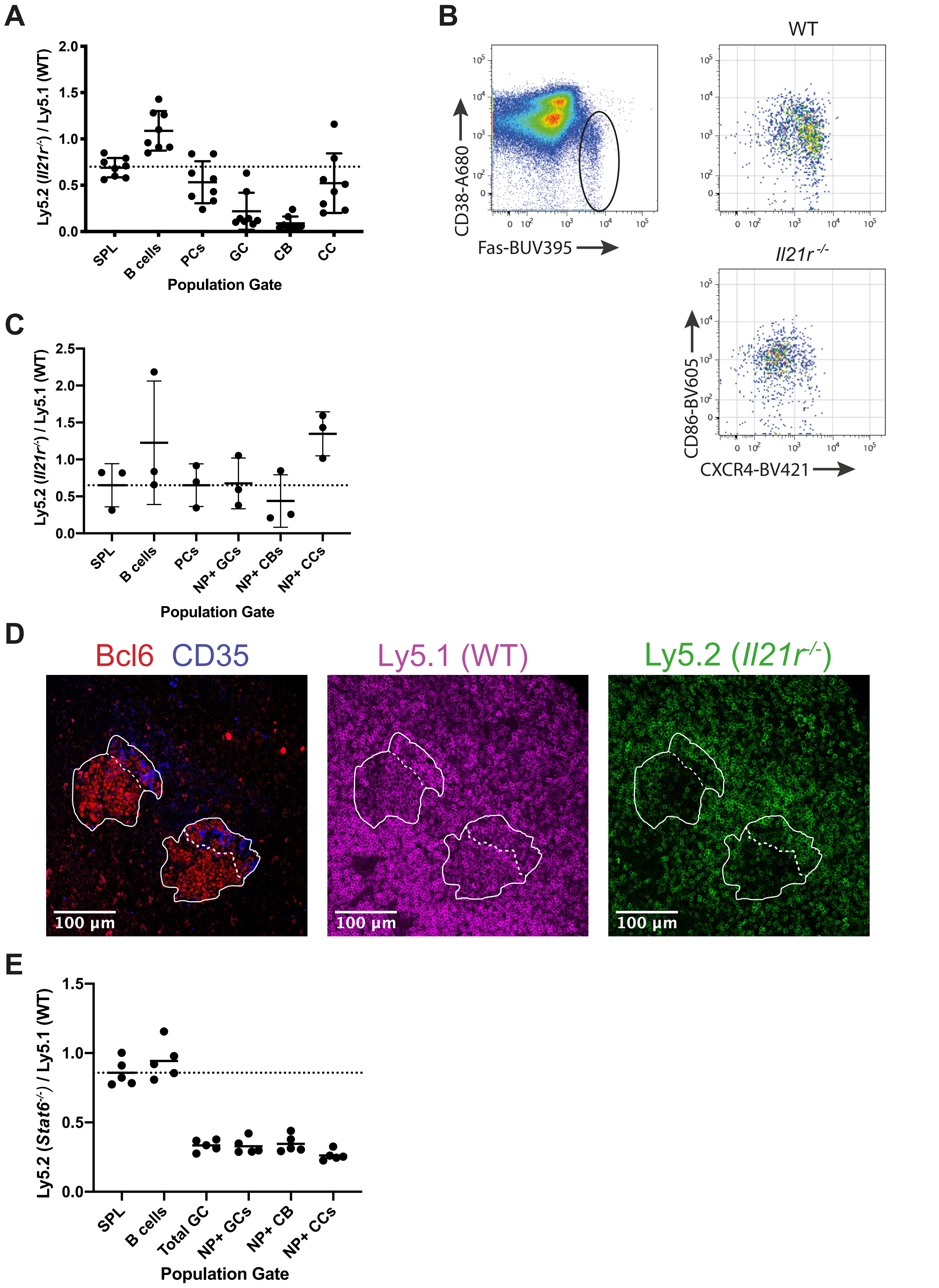


**Supplementary Figure 3. The effects of IL-21 on GC B cells are intrinsic and disintinct from that of IL-4.** (A) and (B) Chimeric mice were generated by bone marrow reconstitution using a 1:1 ratio of WT (Ly5.1) and *Il21r-/-* (Ly5.2) donor cells. Spleens were harvested after reconstitution and analyzed for overall chimerisation and B cell populations. Dotted line represents resulting lymphocyte chimeric ratio in spleen (SPL). (A) Flow cytometry markers used to define populations from unimmunized mice were as follows: B cells: CD19+ PCs: CD138+CXCR4+; GC: CD138-CD19+Fas+CD38-; CB: GC markers and CD86loCXCR4hi; and CC: GC markers and CD86hiCXCR4lo. Ratio calculated as Ly5.2/Ly5.1 with each circle representing an individual mouse (total of n=8) with mean ± SD overlayed. (B) Representative flow cytometry gating, for data shown in (A), from chimeric mice of GC B cells (left) and WT (Ly5.1) and *Il21r-/-* (Ly5.2) zone profiles (middle and right). (C) Flow cytometry markers used to defined splenic populations in mice at d7 post immunization with NP-KLH in alum ip, as outlined in (A). Ratio calculated as Ly5.2/Ly5.1 with each circle representing an individual mouse (total n=3) with mean ±SD overlayed. (A) and (C) represent independent experiments. (D) Representative confocal microscopy staining of spleen section from chimeric mice, d7 after immunization. Left: Bcl6 (red) highlights GC structures and CD35 (blue) highlights FDC structures and LZ; Middle: Ly5.1 (purple) shows WT cells; Right: Ly5.2 (green) shows *Il21r-/-* cells. Solid line defines GC border and dotted line separates LZ from DZ, with CD35 staining only in LZ. Data are repsentative of 3 independent experiments. (E) Proportional representation of WT (Ly5.1) and *Stat6-/-* (Ly5.2) B cells in bone marrow chimeras, d7 after immunization with NP-KLH amongst splenocytes (SPL) and B cells (dashed line showing relative reconstitution), total GC, antigen-specific GC (NP+ GC) and antigen-specific zones (NP+ CB; NP+ CC). Data representative of 2 experiments, with 5 mice shown.

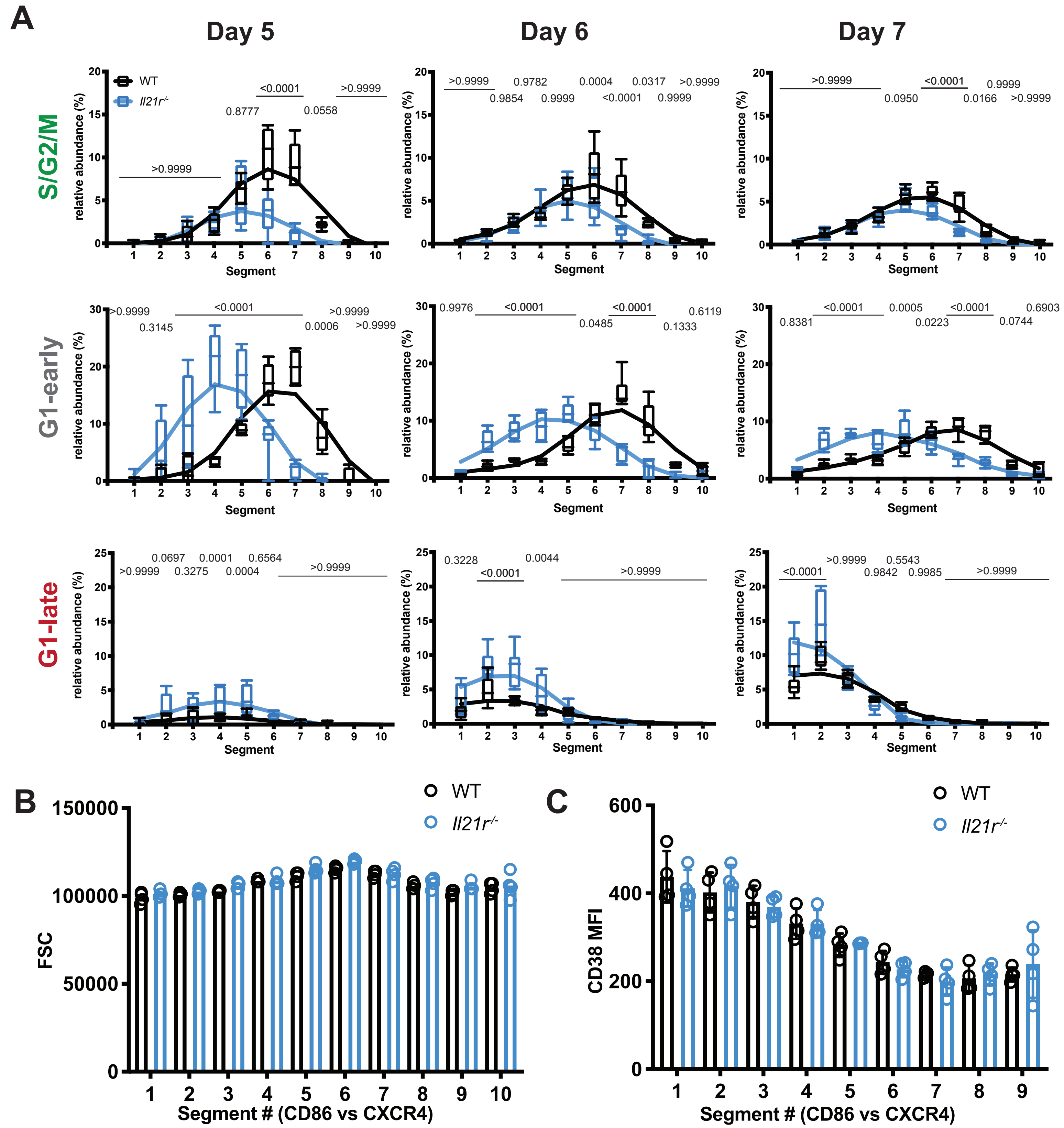


**Supplementary Figure 4. The flow cytometry gating strategy for analysis of antigen-specific splenocytes d7 after immunization of WT and Il21r-/- mice, as detailed in Figure 3**. (A) Box and whisker plots of the relative abundance of cells in individual cell cycle phases among the segments indicated in Figure 3 (A) and (B) at d5, 6 and 7 post immunization. Curves were interpolated using Graph Pad Prism 7 and are as depicted in Figure 3 (C). Data represent n=7, 8 (WT, *Il21r-/-*; Day 5), n=6, 7 (WT, *Il21r-/-*; Day 6) and n=6,8 (WT, *Il21r-/-*; Day 7) mice examined over 3 independent experiments, final gates with ≤10 events were excluded and not counted in n number stated here. Boxes depict 25th to 75th percentiles with the line showing the median. Whiskers show minimum and maximum values. Statistical analysis 2way ANOVA. Exact p-values are shown with those ≤ 0.05 considered significant. (B) and (C) The mean forward scatter (FSC) and CD38 surface mean fluorescence intensity (MFI) are shown for each of the GC segments depicted in Figure 3, dots represent individual mice with mean ± SD indicated by bar and lines respectively. Representative of 2 experiments, and 4 mice per genotype.

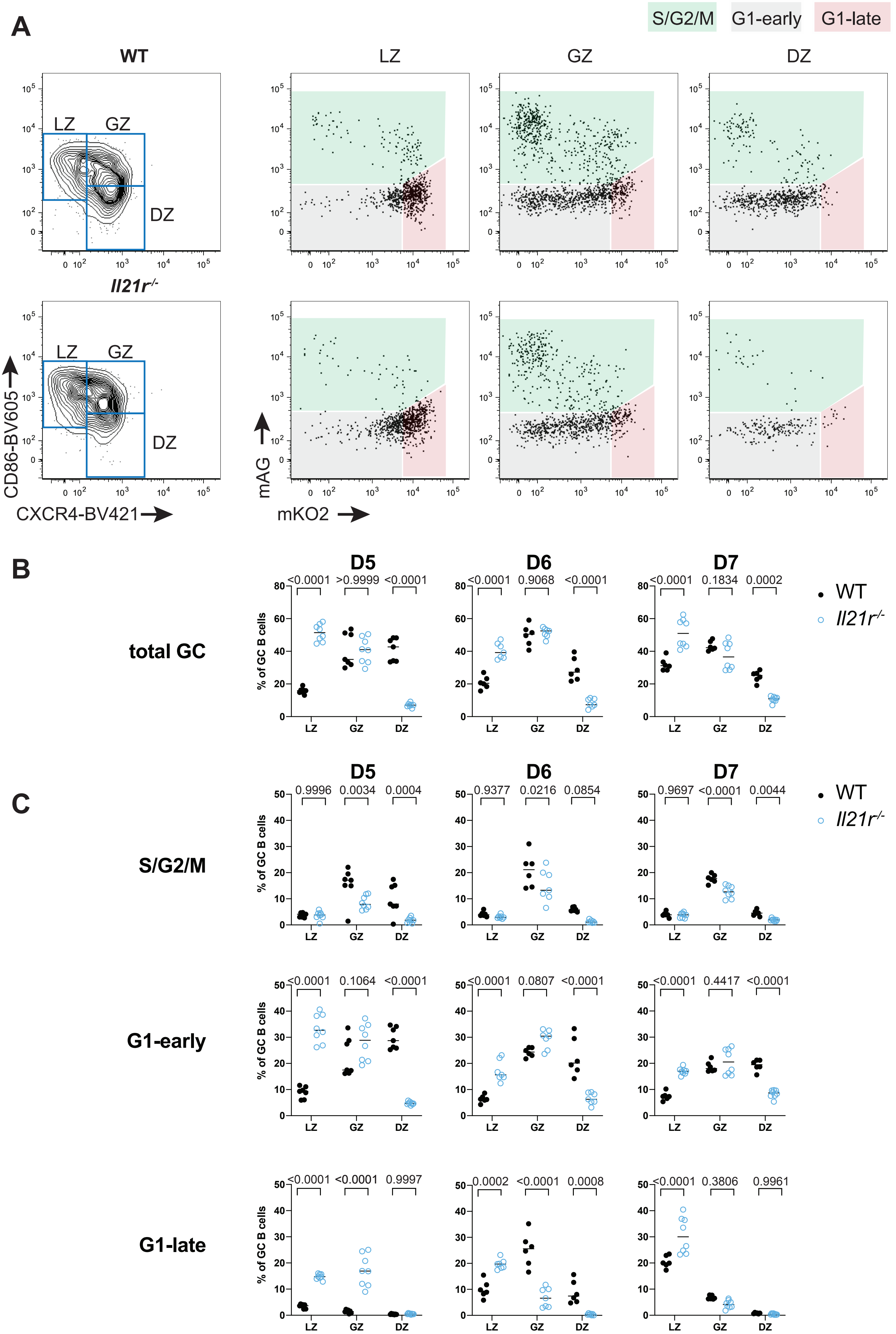


**Supplementary Figure 5. Gating strategy for flow cytometry analysis of data shown in Figure 3 but using LZ, GZ and DZ cells.** (A) Electronic gates used to identify the GC zones and representative flow cytometry plots showing cell cycle distribution in WT and *Il21r-/-* mice within LZ, GZ and DZ. Green, red and grey indicate S/G2/M, G1-early, and G1-late stage cells respectively. (B) Distribution of GC B cells in LZ, GZ and DZ in WT and *Il21r-/-* mice on d5, 6 and 7 post immunization and (C) the proportion each cell cycle zone within zones comprises of the total GC B cell pool. Statistical significance was determined by 2way ANOVA and Sidak's multiple comparisons test. Exact p-values are shown with those ≤ 0.05 considered significant. Data represent n=7, 8 (WT, *Il21r-/-*; Day 5), n=6, 7 (WT, *Il21r-/-*; Day 6) and n=6,8 (WT, *Il21r-/-*; Day 7) examined over 3 independent experiments.

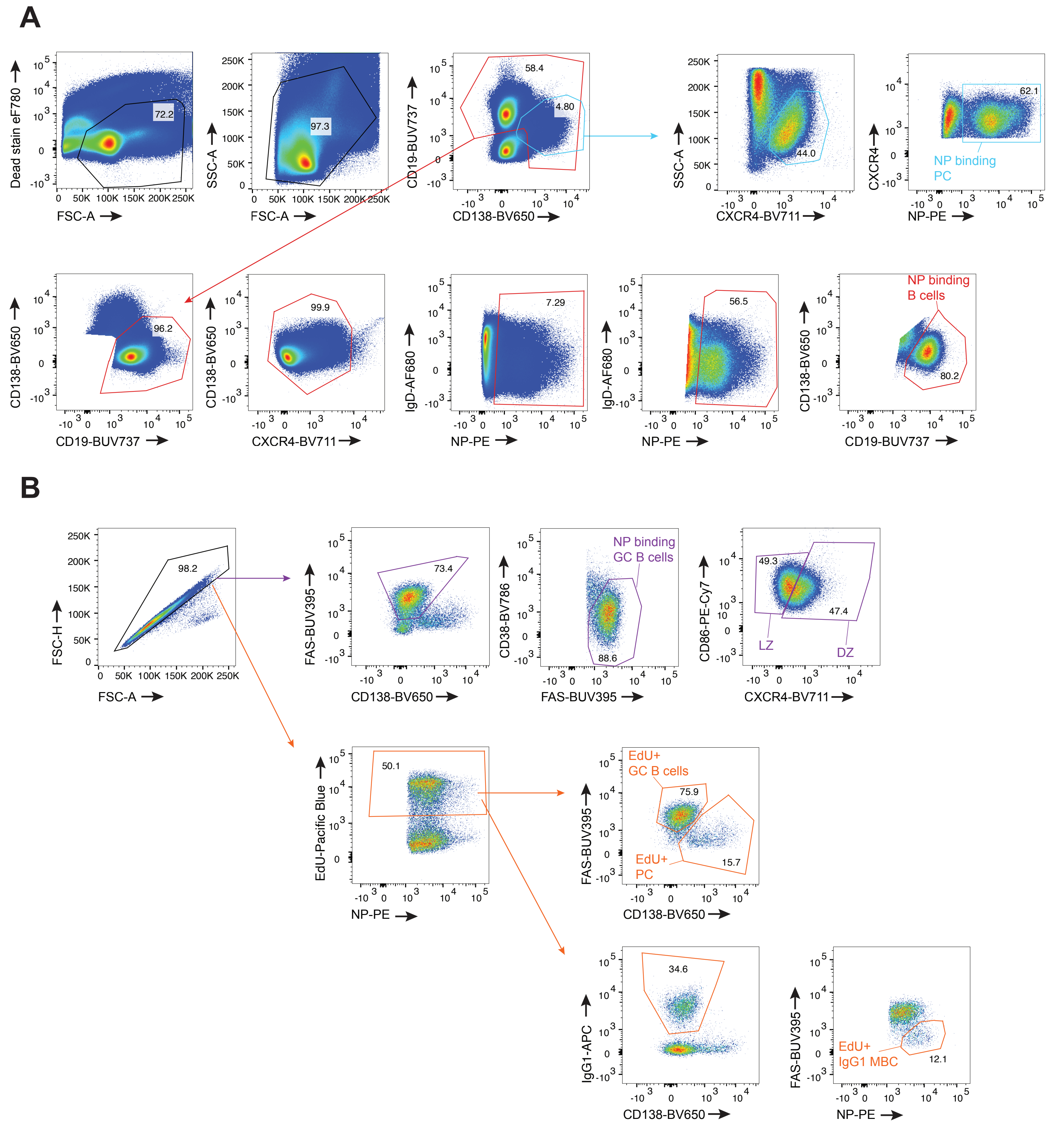


**Supplementary Figure 6. Representative electronic gating strategy as applied in Figure 4.** (A) Black gates are common for all cell types, light blue gates were used to identify NP binding PC and red gates to identify NP binding B cells. (B) NP binding PC and NP binding B cells were then concatenated and exported for further analysis. Gates in purple was used to identify LZ and DZ GC B cells. This gating strategy, in conjunction with EdU and BrdU gates shown in Figure 4B, was used for the analysis shown in Figure 4C-E. Gates in orange depict strategy for Figure 4F, identifying EdU+ GC B cells, PC and IgG1 MBC.

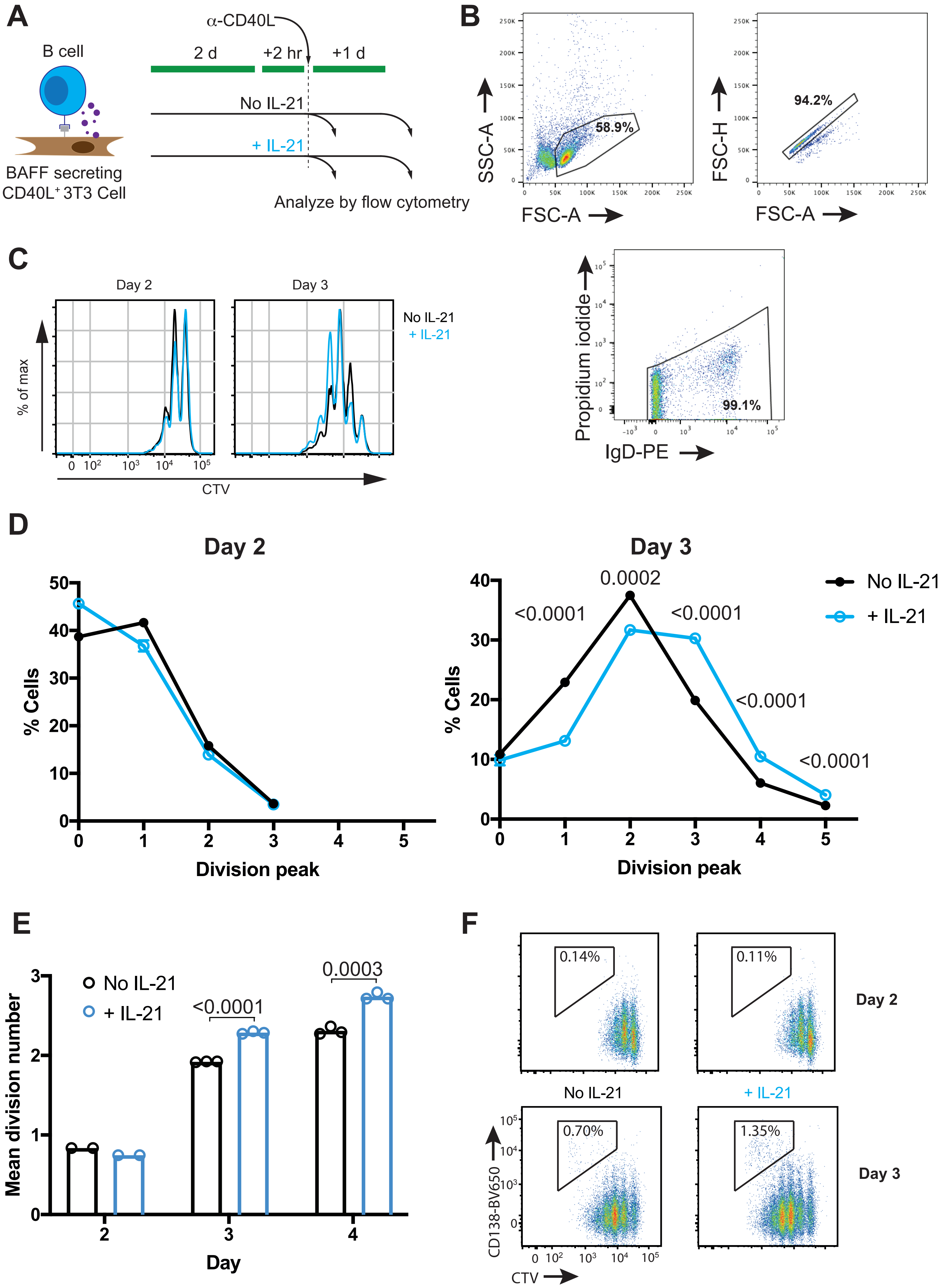


**Supplementary Figure 7. IL-21 promotes cell division progression in iGC B cell cultures.** (A) Experimental setup of in vitro cultured CTV-labelled naïve B cells with CD40L expressing and BAFF secreting feeder cells (α-CD40L is MR-1), pulsed or not on day 2 with IL-21 for 2h, washed then replated without cytokine or CD40 stimulation. (B) Representative flow cytometery electronic gates used to isolate lymphocytes that are live in cultures and applied prior to all data analysed in this figure. (C) Representative flow cytometry of CTV division progression of B cells cultures analyzed at the time points indicated, stimulated for 2 hours without or with IL-21 cytokine. (D) The proportion of B cells per division calculated by CTV dilution is shown at the time points indicated. Stimulation with IL-21 cytokine for 2h is shown in blue. (E) Mean division number was calculated for each stimulation condition at the time point as indicated. (F) Representative flow cytometry gating and frequency of plasmablasts (CD138+) at time points indicated without and with IL-21 cytokine. Data shown were from 1 of 2 independent experiment and represent duplicate (n=2, d2) or triplicate wells (n=3, d3 and 4), mean ±SD. Statistical significance determined with t-test and exact p-values are shown with those ≤ 0.05 considered significant.

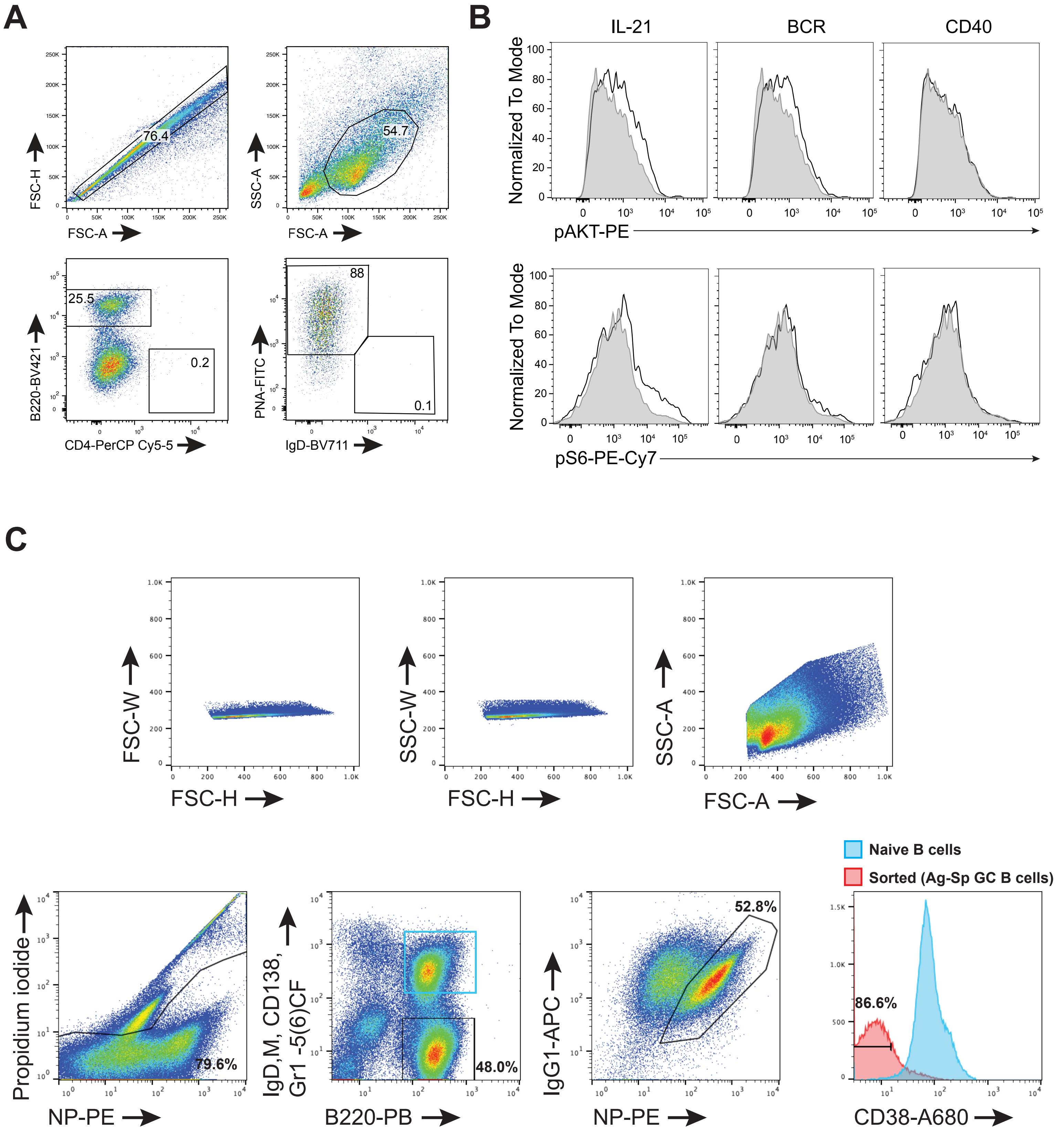


**Supplementary Figure 8. Flow cytometry gating strategies to identify and analyse specific cell types.** (A) Gating strategy to identify GC B cells following MACS enrichment and ex vivo cell culture for phosphoflow analysis. (B) Exemplary histograms showing AKT and S6 phosphorylation of unstimulated GC B cells (grey shaded) or following IL-21, BCR or CD40 stimulation (open histograms). Representative of samples from 3 independent mice of each genotype from 2 independent experiments. (C) Gating strategy applied following MACS enrichment of IgG1+ cells from 3 each of WT and *Il21r-/-* mice on d7 post immunization ip with NP-KLH in alum. Spleen cells that were NP-binding and GC phenotype (CD138-IgM-IgD-Gr1-B220+NP+IgG1+CD38-; red histogram in (C)) were sorted and RNA-seq performed (Figure 5).
