# Supplementary material for: The concerted change in the distribution of cell cycle phases and zone composition in germinal centers is regulated by IL-21
Source: Nat Commun. 2021 Dec 9;12:7160. doi: 10.1038/s41467-021-27477-0 (PMC8660905; doi:10.1038/s41467-021-27477-0)
Supplement: Supplementary file 2 — Reporting Summary [file 41467_2021_27477_MOESM2_ESM.pdf]

nature research

Corresponding author(s): David Tarlinton

Last updated by author(s): Oct 20, 2021

Reporting Summary

Nature Research wishes to improve the reproducibility of the work that we publish. This form provides structure for consistency and transparency in reporting. For further information on Nature Research policies, see our Editorial Policies and the Editorial Policy Checklist.

Statistics

For all statistical analyses, confirm that the following items are present in the figure legend, table legend, main text, or Methods section.

n/a	Confirmed	
☐	☒	The exact sample size (*n*) for each experimental group/condition, given as a discrete number and unit of measurement
☐	☒	A statement on whether measurements were taken from distinct samples or whether the same sample was measured repeatedly
☐	☒	The statistical test(s) used AND whether they are one- or two-sided<br>*Only common tests should be described solely by name; describe more complex techniques in the Methods section.*
☒	☐	A description of all covariates tested
☐	☒	A description of any assumptions or corrections, such as tests of normality and adjustment for multiple comparisons
☐	☒	A full description of the statistical parameters including central tendency (e.g. means) or other basic estimates (e.g. regression coefficient) AND variation (e.g. standard deviation) or associated estimates of uncertainty (e.g. confidence intervals)
☐	☒	For null hypothesis testing, the test statistic (e.g. *F*, *t*, *r*) with confidence intervals, effect sizes, degrees of freedom and *P* value noted<br>*Give P values as exact values whenever suitable.*
☒	☐	For Bayesian analysis, information on the choice of priors and Markov chain Monte Carlo settings
☒	☐	For hierarchical and complex designs, identification of the appropriate level for tests and full reporting of outcomes
☒	☐	Estimates of effect sizes (e.g. Cohen's *d*, Pearson's *r*), indicating how they were calculated

Our web collection on statistics for biologists contains articles on many of the points above.

Software and code

Policy information about availability of computer code

Data collection	BD FACS Diva, Illumina CASAVA pipeline 1.8.2 HiSeq 2000
Data analysis	Graph Pad Prism 7 & 8, FlowJo v9.9.6 and 10, Rsubread, Limma package 3.16.8, EdgeR 4.0.34

For manuscripts utilizing custom algorithms or software that are central to the research but not yet described in published literature, software must be made available to editors and reviewers. We strongly encourage code deposition in a community repository (e.g. GitHub). See the Nature Research guidelines for submitting code & software for further information.

Data

Policy information about availability of data

All manuscripts must include a data availability statement. This statement should provide the following information, where applicable:

- Accession codes, unique identifiers, or web links for publicly available datasets
- A list of figures that have associated raw data
- A description of any restrictions on data availability

RNAseq data have been submitted to GEO with accession number GSE184475 [https://www.ncbi.nlm.nih.gov/geo/query/acc.cgi?acc=GSE184475]. All other relevant data are available from the corresponding author on reasonable request.

Field-specific reporting

Please select the one below that is the best fit for your research. If you are not sure, read the appropriate sections before making your selection.

☒ Life sciences ☐ Behavioural & social sciences ☐ Ecological, evolutionary & environmental sciences

For a reference copy of the document with all sections, see nature.com/documents/nr-reporting-summary-flat.pdf

Life sciences study design

All studies must disclose on these points even when the disclosure is negative.

Sample size	The sample size was not pre-determined and no formal power calculation was performed. However, for all experiments, at least 3 mice per genotype/time point were included and all experiments were performed at least twice (except for Fig 4 and Supp Fig 2). The number of mice was chosen to perform statistical tests and derive statistical significance as indicated in the figure legends.
Data exclusions	Mice that did not response to immunisation were excluded from the ananlysis, non-responding mice were determined by negative day 5 NP-specific IgM serum titre. Data points were excluded from flow cytometery analysis if the number of events in a gate for analysis was <10, this has been clearly indicated in the methods and figure legends where this occurred.
Replication	All experiments conducted and described have been replicated at least once (refer to figure legends for exact repeat number), with the exception of fig 4 and Supp Fig 2. All attempts of replication were successful and included in figures provided, either pooled in figures or separated.
Randomization	All experimental samples were partitioned based on genotype. Covariates were controlled by matching for age, sex and co-housed to ensure non-bias approach.
Blinding	Blinding was not possible, in order to ensure enough mice of each genotype and that they were age and sex matched, researchers were informed of the mouse groups. Additionally, to ensure equal numbers of mice per group at different time points, researchers were informed and not blinded to groups. However measurements shown from this study were objectively quantified (e.g. % of population by FACS), thus blinding would not have changed this.

Reporting for specific materials, systems and methods

We require information from authors about some types of materials, experimental systems and methods used in many studies. Here, indicate whether each material, system or method listed is relevant to your study. If you are not sure if a list item applies to your research, read the appropriate section before selecting a response.

Materials & experimental systems

n/a	Involved in the study	
☐	☒	Antibodies
☐	☒	Eukaryotic cell lines
☒	☐	Palaeontology and archaeology
☐	☒	Animals and other organisms
☒	☐	Human research participants
☒	☐	Clinical data
☒	☐	Dual use research of concern

Methods

n/a	Involved in the study	
☒	☐	ChIP-seq
☐	☒	Flow cytometry
☒	☐	MRI-based neuroimaging

Antibodies

Antibodies used

B220-AF647 (clone RA3-6B2, in house purified and conjugated to Alexa fluor 647)
B220-BV421 (clone RA3-B62, BD Biosciences, Cat. 562922; 1 in 200 dilution),
B220-PB (clone RA3-6B2, BD Biosciences, Cat. 558108; 1 in 200 dilution)
Bcl6-CF568 (clone 7D1-10, in house purified and conjugated to CF568 (Biotium, Cat. 92215); 1 in 200 dilution),
biotin microbeads (Miltenyi Biotec, Cat. 130-090-485; 1 in 200 dilution)
BrdU-FITC (FITC BrdU Flow Kit, BD Biosciences, Cat. 559619; 1 in 200 dilution),
CD138 (clone 281-2, BD Biosciences, Cat. 553713; 1 in 200 dilution)
CD138-5(6)CF (clone 281; in house generated; 1 in 200 dilution),
CD138-APC (clone 281.2, BD Biosciences, Cat. 561705; 1 in 200 dilution)
CD138-BV650 (clone 281.2, BD Biosciences, Cat. 564068; 1 in 200 dilution)
CD16/32 (clone 2.4G2, in-house hybridoma; 1 in 50 dilution),
CD19-APC (clone 1D3, BD Biosciences, Cat. 550992; 1 in 200 dilution),
CD19-BUV395 (clone 1D3, BD Biosciences, Cat. 563557; 1 in 200 dilution),
CD19-BUV737 (clone 1D3, BD Biosciences, Cat. 564296; 1 in 200 dilution),
CD19-PE (clone 1D3, eBioscience, Cat. 12-0193-83; 1 in 200 dilution),

CD19-PECy7 (clone 1D3, eBioscience, Cat. 25-0193-82; 1 in 200 dilution),
CD35-biotin (BD Biosciences, Cat. 553816; 1 in 200 dilution),
CD38-A680 (clone NIMR5, in house generated; 1 in 200 dilution),
CD38-BV786 (clone Ab90, BD Biosciences, Cat. 740887; 1 in 200 dilution),
CD4 (clone GK1.5, WEHI Antibody Facility; 1 in 200 dilution),
CD4-A680 (clone GK1.5; 1 in 200 dilution),
CD4-PerCP-Cy5.5 (clone RM4-5, BD Biosciences, Cat. 550954; 1 in 200 dilution)
CD44-APC (clone IM7, BD BrdU, BD Biosciences, Cat. 559250; 1 in 200 dilution),
CD45.1-eFluor450 (clone A20, eBioscience, Cat. 48-0453-82; 1 in 200 dilution),
CD45.2-FITC (clone 104, BD Biosciecnes, Cat. 553772; 1 in 200 dilution)
CD62L-PB/V450 (clone MEL-14, BD Biosciences, Cat. 560507; 1 in 200 dilution),
CD8 (clone YTS.169, WEHI Antibody Facility; 1 in 200 dilution)
CD86-BV605 (clone GL1, BD Biosciences, Cat.563055; 1 in 200 dilution),
CD86-PE-Cy7 (clone GL1, BD Biosciences, Cat. 560582; 1 in 200 dilution),
CD95-BUV395 (clone Jo2, BD Biosciences, Cat. 740254; 1 in 200 dilution),
CXCR4-BV421 (clone 2B11, BD Biosciences, Cat. 562738; 1 in 50 dilution),
CXCR4-BV711 (clone 2B11, BD Biosciences, Cat. 740734; 1 in 50 dilution).
CXCR5-Biotin (clone 2G8, BD Biosciences, Cat. 551960; 1 in 50 dilution),
Fas-BUV395 (clone Jo2, BD Biosciences, Cat. 740254; 1 in 200 dilution),
Fas-PECy7 (clone Jo2, BD Biosciences, Cat. 557653; 1 in 200 dilution),
GR1 (clone 8C5, WEHI Antibody Facility; 1 in 200 dilution),
Gr1-5(6)CF (clone Rb6-8C5; in house generated; 1 in 200 dilution),
Gr1-Biotin (clone Rb6-8C5; 1 in 200 dilution),
IgD (clone 11-26, Southern Biotech, Cat. 1120-08; 1 in 200 dilution),
IgD-5(6)CF (clone 11-26C; in house generated; 1 in 200 dilution),
IgD-Alexa FluorTM680 (clone 11-26c.2a, in-house hybridoma and conjugated using Alexa Fluor™ 680 NHS Ester, ThermoFischer Scientific, Cat. A20008; 1 in 200 dilution),
IgD-BV711 (clone 11-26c.2a, BD Biosciences, Cat. 564275; 1 in 200 dilution),
IgD-PE (clone 1126.2a, Southern Biotec, Cat. 1120-09; 1 in 200 dilution),
IgG1 (Miltenyi Biotec, Cat. 130-047-101; 1 in 200 dilution),
IgG1-APC (clone X56, BD Biosciences, Cat. 550874; 1 in 200 dilution),
IgM-5(6)CF (clone 331.12; in house generated; 1 in 200 dilution),
IgM-Biotin (clone 331.12; 1 in 200 dilution),
IgM-BV510 (clone R6-60.2, BD Biosciences, Cat. 563118; 1 in 200 dilution),
Ki-67-PE (Clone 11F6, BioLegend cat. #151209; 1 in 100 dilution),
Ly5.1-PECy7 (clone A20, eBioscience, Cat. 25-0453-82; 1 in 200 dilution),
Ly5.1-PerCPCy5.5 (clone A20, eBioscience, Cat. 45-0453-80; 1 in 200 dilution),
Ly5.2-FITC (clone 104, BD Biosciences, Cat. 553772; 1 in 200 dilution),
NP conjugated PE (in-house conjugated; 1 in 200 dilution),
NP-APC (NIP-CAP-OSu, Biosearch Technologies, Cat. N-1110-100; 1 in 200 dilution),
NP-PE (NIP-CAP-OSu, Biosearch Technologies Cat. N-1110-100 and ThermoFisher Scientific, Cat. P801; in house conjugated; 1 in 200 dilution),
p-S6 (Ser235/236)-PE-Cy7 (clone D57.2.2E, Cell Signaling, Cat. 34411S; 1 in 100 dilution),
pAKT (S473)-PE (clone M89-61, BD Biosciences, Cat. 561671; 1 in 40 dilution),
PD1-PECy7 (clone RMP-30, Biolegend, Cat. 109110; 1 in 200 dilution),
PNA-FITC (Vector Laboratories, Cat. FL-1071; 1 in 200 dilution),
StAv-BUV737 (BD Biosciences, Cat. 564293; 1 in 200 dilution),
StAv-BV650 (clone BD 5, Biosciences, Cat. 63855; 1 in 200 dilution),

Validation

Antibody validation relied on the manufacturers statements as provided on their respective websites.

Eukaryotic cell lines

Policy information about cell lines

Cell line source(s)

40LB (Nojima et al, Nat Comms, 2011)
Daisuke Kitamura, MD. PhD.
Research Institute for Biomedical Sciences,
Tokyo University of Science
Yamazaki 2669, Noda, Chiba 278-0022, Japan

Authentication

Cell line were distinguished based on morphology under microscope and their function (ie. differentiations of B cells).

Mycoplasma contamination

This line was not tested for mycoplasma contamination.

Commonly misidentified lines
(See ICLAC register)

This line is not commonly misidentified.

Animals and other organisms

Policy information about studies involving animals; ARRIVE guidelines recommended for reporting animal research

Laboratory animals

All mice were bred and maintained under specific pathogen-free conditions at the Walter and Eliza Hall Institute or Monash

Laboratory animals	University Animal Research Platform facility. C57Bl/6, Ly5.1 (or Ly5.2), IL21R-/-, IL21-/- , FUCCI (WT or IL21R-/-) and STAT6-/- mice were age and sex matched for all experiments, mice were aged between 6 to 26 weeks. Both female and male mice were used for experiments. The mice were housed at an ambient temperature of 20-22oC, and they were kept under a 14 hours day and 10 hours night cycle.
Wild animals	This study did not involve wild animals.
Field-collected samples	This study did not involve samples collected from the field.
Ethics oversight	All the animal studies describes in this manuscript followed the guidelines and protocols approved by either the Walter and Eliza Hall Institute Animal Ethics Committee or the Alfred Research Alliance Animal Ethics Committee.

Note that full information on the approval of the study protocol must also be provided in the manuscript.

Flow Cytometry

Plots

Confirm that:

☒ The axis labels state the marker and fluorochrome used (e.g. CD4-FITC).

☒ The axis scales are clearly visible. Include numbers along axes only for bottom left plot of group (a 'group' is an analysis of identical markers).

☒ All plots are contour plots with outliers or pseudocolor plots.

☒ A numerical value for number of cells or percentage (with statistics) is provided.

Methodology

Sample preparation	Spleens were harvested from mice and collected in PBS+2%FCS. Single cells suspensions were obtained by pushing the organ through a 70um sieve with a syringe plunger. The blood were removed with red cell removal buffer (generated in house; 1-2 min incubation followed by washing) or via acid treatments or via magnetic beads, as described in detail in the methods of manuscript. All samples were filtered through 70um mesh prior to staining for flow cytometry and prior to running on the instrument for acquisition.
Instrument	Flow cytometry analysis was performed on a BD Fortessa x20 (BD Biosciences). Cell sorting was performed on a BD Influx or BD FACS-Aria II/III (BD Biosciences).
Software	The collection of the data was done with BD FACS Diva on the above instruments, analysis of flow cytometry data was done using FlowJo v9.9.6 and v10.
Cell population abundance	Purity of sorted samples for RNASeq was assessed after each sort b reanalysis on the instrument, the purity of all sorts was >99%.
Gating strategy	The cells were firstly gated on size using SSC-A/FSC-A, then on single cells based on FSC-H/FSC-A and then on live cell gate based on viability stain used (SYTOX Blue, FluoroGold, PI). All gates subsequent to live cells are described and/or exemplified in the relevant sections of the manuscript.

☒ Tick this box to confirm that a figure exemplifying the gating strategy is provided in the Supplementary Information.
